# Clinical and molecular response to alpha1‐oleate treatment in patients with bladder cancer

**DOI:** 10.1002/cam4.70149

**Published:** 2024-09-10

**Authors:** Farhan Haq, Samudra Sabari, Jaromir Háček, Antonín Brisuda, Ines Ambite, Michele Cavalera, Parisa Esmaeili, Murphy Lam Yim Wan, Shahram Ahmadi, Marek Babjuk, Catharina Svanborg

**Affiliations:** ^1^ Division of Microbiology, Immunology and Glycobiology, Department of Laboratory Medicine, Faculty of Medicine Lund University Sweden; ^2^ Department of Pathology and Molecular Medicine Motol University Hospital, 2nd Faculty of Medicine, Charles University Praha Prague Czech Republic; ^3^ Department of Urology Motol University Hospital, 2nd Faculty of Medicine, Charles University Praha Prague Czech Republic

**Keywords:** alpha1‐oleate complex, cell shedding, cellular uptake, gene expression, non‐muscle invasive bladder cancer

## Abstract

**Background:**

The tumoricidal complex alpha1‐oleate targets bladder cancer cells, triggering rapid, apoptosis‐like tumor cell death. Clinical effects of alpha1‐oleate were recently observed in patients with non‐muscle invasive bladder cancer (NMIBC), using a randomized, placebo‐controlled study protocol.

**Aims:**

To investigate if there are dose‐dependent effects of alpha1‐oleate.

**Materials and Methods:**

Here, patients with NMIBC were treated by intravesical instillation of increasing concentrations of alpha1‐oleate (1.7, 8.5, or 17 mM) and the treatment response was defined relative to a placebo group.

**Results:**

Strong, dose‐dependent anti‐tumor effects were detected in alpha1‐oleate treated patients for a combination of molecular and clinical indicators; a complete or partial response was detected in 88% of tumors treated with 8.5 mM compared to 47% of tumors treated with 1.7 mM of alpha1‐oleate. Uptake of alpha1‐oleate by the tumor triggered rapid shedding of tumor cells into the urine and cell death by an apoptosis‐like mechanism. RNA sequencing of tissue biopsies confirmed the activation of apoptotic cell death and strong inhibition of cancer gene networks, including bladder cancer related genes. Drug‐related side effects were not recorded, except for local irritation at the site of instillation.

**Discussion and Conclusions:**

These dose‐dependent anti‐tumor effects of alpha1‐oleate are promising and support the potential of alpha1‐oleate treatment in patients with NMIBC.

## 1 INTRODUCTION

Bladder cancer has been identified as an unmet medical need, as most current therapies are suboptimal.^1^ Recurrence rates are high after surgical tumor resection and the limited efficacy and side effects of chemotherapy, and immunotherapy remain a major concern.^2^, ^3^, ^4^, ^5^, ^6^, ^7^, ^8^ The peptide‐fatty acid complex alpha1‐oleate is an investigational new drug that kills tumor cells of different origins.^9^, ^10^, ^11^, ^12^ Therapeutic efficacy of its parent molecule HAMLET (Human Alpha‐lactalbumin Made LEthal to Tumor cells) has been documented in animal models of colon cancer, bladder cancer and glioblastoma, and clinical effects have been reported in patients with bladder cancer or skin papillomas.^12^, ^13^, ^14^, ^15^, ^16^, ^17^ Intravesical instillation of HAMLET triggered rapid tumor cell shedding in patients with non‐muscle invasive bladder cancer (NMIBC), suggesting direct effects on the tumors.^14^

The alpha1‐oleate complex formed by the N‐terminal peptide of alpha‐lactalbumin and oleic acid. The synthetic peptide‐based complex reproduces the tumoricidal effects of HAMLET and has shown therapeutic efficacy in a randomized, placebo‐controlled clinical trial of NMIBC patients, without drug related side effects.^18^, ^19^ Alpha1‐oleate treatment (1.7 mM) was shown to reduce tumor size, trigger rapid tumor cell shedding and induce tumor cell death in NMIBC patients.^19^ In addition, alpha1‐oleate was shown to trigger a local immune response similar to BCG,^20^ suggesting a dual mechanism of action by directly killing tumor cells and activating an immune response with anti‐tumor properties.

A dose‐dependent increase in therapeutic efficacy at higher doses was detected in the MB49 murine bladder cancer model, using 1.7, 8.5 or 17 mM of alpha1‐oleate for intravesical instillation.^17^ Bladder cancer progression was delayed long‐term at the 8.5 mM dose and at the highest dose, treated bladders remained similar to those in healthy mice, with no evidence of tumor progression.^17^ The clinical trial program was therefore extended with a dose‐escalation part, using the same primary and secondary end points to define treatment outcomes as in the placebo‐controlled part of the study. The anti‐tumor effects increased significantly in patients receiving 8.5 mM of alpha1‐oleate, compared to the lower dose (1.7 mM) or placebo and cancer‐related gene expression was strongly inhibited, including gene networks controlling the tumor environment, tumor invasion and metastasis. The dose‐dependent increase treatment effect of alpha1‐oleate confirms its therapeutic potential in patients with NMIBC. The complete and partial responses of the tumors suggest that the rapid effect of alpha1‐oleate might improve the precision of subsequent surgery.

## 2 MATERIALS AND METHODS

### 2.1 Trial design and patient population

The single center trial of alpha1‐oleate in patients with NMIBC (EudraCTNo:2016–004269‐14, ClinicalTrials.gov NCT 03560479) was amended with a dose‐finding part, using the same eligibility criteria, schedule of visits, study treatment administration, primary and secondary end‐points and assessments as the first part of the study.^19^ Patients in the first part of the study received intravesical instillations of alpha1‐oleate (1.7 mM, *n* = 20) or placebo (*n* = 20) on six occasions, during 1 month preceding scheduled transurethral resection of bladder tumor (TURBT) and all patients completed the study (CONSORT flow chart in Figure S1). Patients in the dose‐finding part received a five times higher dose (8.5 mM, *n* = 6) or a 10‐times higher dose (17 mM, *n* = 3) of alpha1‐oleate. All patients receiving 8.5 mM of alpha1‐oleate completed the treatment, as did 2/3 of the patients receiving 17 mM of alpha1‐oleate. Primary end points were safety, change in tumor size and tumor cell shedding into the urine. Secondary end points were histopathology evaluation of tumor biopsies, quantification of alpha1‐oleate uptake by the tumor, apoptosis induction and treatment effects on gene expression.

Subjects diagnosed with NMIBC and scheduled for transurethral surgery were included in the study. The study was approved by the State Institute for Drug Control (SUKL) in the Czech Republic; number 273799/17‐I and the Ethics Committee of the Motol University Hospital; number EK‐786/17. All patients were included in the study after informed consent. The dose escalation part of the study was an extension of the single center, placebo controlled, double blinded randomized Phase I/II interventional clinical trial of NMIBC (EudraCT Number: 2016–004269‐14 and ClinicalTrials.gov NCT03560479). Demographic data, morbidity and health parameters as well as tumor characteristics were recorded by the study physicians in the electronic Case Report Form (eCRF) and closely monitored by an external monitor.

### 2.2 GMP peptide synthesis and alpha1‐oleate formulation

The synthesis of the drug substance and the production of the investigational drug product were in compliance with good manufacturing practice (GMP) guidelines. Fmoc solid phase chemistry was used to synthesize the 39 amino acid peptide (aa 1–39), Ac‐KQFTKAELSQLLKDIDGYGGIALPELIATMFHTSGYDTQ‐OH, achieving >95% purity (Polypeptide group, France). To form the alpha1‐oleate complexes, the peptide was mixed with sodium oleate at a 1:5 molar ratio. The complex was diluted to the intended concentrations of 1.7, 8.5, or 17 mM in phosphate‐buffered saline (PBS) and dispensed into bottles for clinical use (Rechon Malmö, Sweden). The bottles were stored at −20°C until use. The placebo cohort received a PBS solution that was, visually indistinguishable from the active treatment.

### 2.3 Study protocol

This study evaluated the safety and efficacy of intravesical instillation of alpha1‐oleate in subjects with NMIBC. Patients were diagnosed based on cystoscopy appearance of the tumor and were on the waiting list for TURBT. After informed consent, the subjects were enrolled and received intravesical instillations of either alpha1‐oleate or placebo on six occasions during 1 month preceding TURBT. A safety follow‐up was performed 52 days after the first instillation. The study underwent data lock and subsequent unblinding was under third‐party control. The placebo‐controlled part of the study was extended with a dose‐escalation part.

### 2.4 Inclusion criteria

1. Patient with non‐muscle invasive papillary bladder cancer (NMIBC) based on cystoscopy appearance, on the waiting list for TURB.
2. Negative pregnancy test in women of childbearing potential.
3. Appropriate methods of contraception in women of childbearing potential during study.
4. Patients should be able to keep the content of the bladder for at least 1 hour.

### 2.5 Exclusion criteria

1. Patient with a previous history of muscle invasive bladder cancer.
2. Patient with a history of NMIBC with an interval shorter than 6 months after previous TURB.
3. Previous intravesical Bacillus Calmette‐Guerin (BCG) immunotherapy in the last 12 months.
4. Previous intravesical chemotherapy in the last 12 months.
5. Participants with any other cancer diagnosis within the last 5 years (except of skin basaliomas).
6. Acute urinary tract infection.
7. Participants with prior radiotherapy or systemic chemotherapy.
8. Participants receiving any other investigational agent or non‐marketed product 1 month prior to Visit 1 and during the trial.
9. Any concurrent illness that may render a participant ineligible or limit compliance with study requirements.
10. Previously enrolled in this trial.

### 2.6 Primary and secondary endpoints

The primary endpoints include safety as AEs profile, change from baseline in tumor size and characteristics of papillary tumors and cell shedding. Secondary endpoints included histopathology scoring and urine cytology, uptake of alpha1‐oleate by tumor cells and tissues, apoptosis induction in cells, and tumor tissues.

#### 2.6.1 Primary end points

1. Safety as AEs profile (time frame: from signing of informed consent (Day 1) and until end of study (Day 52): incidence of AEs and classification in terms of severity, causality and outcome.
2. Change from baseline in characteristics of papillary tumors (time frame: prior to treatment (baseline) and on Day 30, in connection with scheduled surgery): the bladder tumors are characterized by in vivo imaging during examination by cystoscopy.
3. Efficacy as cell shedding (time frame: Days 1 to 22): change in cell shedding into urine (number of epithelial cells per mL of urine).

#### 2.6.2 Secondary endpoints

1. Histopathology scoring of the tumor using established parameters for scoring of Grade and Stage/Invasiveness.
2. Urine cytology examined before and after instillation, using the Paris scoring system.
3. Uptake of alpha1‐oleate by tumor cells and tumor tissue, defined by staining with specific antibodies.
4. Cellular and tissue apoptotic response to alpha1‐oleate, defined by transferase dUTP nick end‐labelling (TUNEL) staining.
5. Tumor response to alpha1‐oleate, defined by RNA sequence analysis.
6. Long‐term effects of the study treatment have not been evaluated.

### 2.7 Adverse events profile

AEs were documented from the time of informed consent signing until the conclusion of the study (FU1 Visit, day 52). Any diagnoses, symptoms, signs, or findings arising after treatment with the study drug were logged as AEs or severe AEs (SAEs). (S)AEs linked to the study procedure were categorized during the trial using MedDRA terminology by preferred terms and primary system organ class. All AEs noted during the trial were included in subject data listings, and an overall summary was compiled, detailing the number (percentage) of subjects experiencing any treatment‐emergent (S)AEs, premature trial discontinuations due to AEs, treatment‐related AEs, and (S)AEs.

### 2.8 Analysis of urine samples

Urine samples were collected from each patient before and after each instillation of alpha1‐oleate or placebo (from Visit 1 to Visit 6).

#### 2.8.1 Cell shedding

Cell shedding was quantified by light microscopy by counting the total number of epithelial cells in uncentrifuged urine using a hemocytometer chamber. Changes in cell shedding were determined at each visit by comparing cell counts in samples collected before and after each instillation. Additionally, cell clusters were identified based on samples examined by an experienced pathologist, who scored them on a scale of 0–2, where 0 indicated no clusters and 2 represented the highest cluster count.

#### 2.8.2 Urine cytology

Cells in urine were centrifuged onto poly‐L‐lysine coated microscope slides (Cytospin 3, Shandon) at 113×g for 5 min, fixed and stored at room temperature until further analysis. Urinary cytology was assessed using the Paris System for Reporting Urinary Cytology 2016, with classifications as follows: (1) No diagnosis/unsatisfactory. (2) Negative for high‐grade urothelial carcinoma. (3) Presence of atypical urothelial cells. (4) Suspicious for high‐grade urothelial carcinoma. (5) High‐grade urothelial carcinoma. (6) Low‐grade urothelial neoplasm. (7) Other findings positive for malignancies and miscellaneous lesions.

#### 2.8.3 ‐ Alpha1 uptake by immunohistochemistry

Alpha1‐oleate uptake by tumor cells was quantified by staining with specific antibodies. Fixed cells on cytospin slides were washed (PBS, 10 min), permeabilized (0.25% TritonX‐100 in PBS, 20 min, room temperature). Tumor tissue sections were deparaffinized with xylene followed by serial dehydration with ethanol (100%, 95%, 75% and 50%). Dehydrated sections were fixed (4% PFA, 15 min), permeabilized (0.25% TritonX‐100 in PBS, 20 min, room temperature). Slides were then blocked (5% normal goat serum in PBS or TBS, 1 h, room temperature) before addition of rabbit polyclonal anti‐human alpha lactalbumin antibodies (1:50 in 5% normal goat serum at 4°C, overnight, Mybiosource, Cat# MBS175270). Slides were washed (PBS‐T or TBS‐T, 2 × 5 min) and stained with Alexa‐488 labeled secondary antibodies (1:200, 1 h, room temperature, Invitrogen, A11034). The nucleus was counterstained using DAPI (1:1000, 15 min) before a final wash (3 × 5 min in PBS or TBS). Slides were mounted (Fluoromount aqueous mounting media, Sigma, F4680 or Prolong Glass Antifade Mountant, Invitrogen, P36980), before capturing images by laser scanning confocal microscopy (Zeiss LSM 900) or NanoZoomer slide scanner (Hamamatsu Photonics). Fluorescence intensity was quantified by ImageJ and net fluorescence calculated after subtraction of the secondary antibody background.

### 2.9 Apoptosis detected by TUNEL staining

DNA fragmentation was detected using the terminal deoxynucleotidyl TUNEL assay (Click‐iT TUNEL Alexa Fluor 488 imaging assay kit, ThermoFisher, C10245). Fixed cells on cytospin slides were washed (PBS, 10 min) then permeabilized (0.25% TritonX‐100 in PBS, 20 min, room temperature). Tissue sections were deparaffinized with xylene followed by serial dehydration with ethanol (100%, 95%, 75% and 50%). Dehydrated sections were fixed (4% PFA, 15 min), permeabilized (DNase‐free Protease K solution 20 μg/mL, 15 min). Slides were then incubated with TUNEL reaction mixture containing TdT for 60 min at 37°C. After the TUNEL reaction, sections were incubated with Click‐iT reaction mixture (30 min, 37°C). Slides were counterstained with DAPI (1 μg/mL, 5 min), mounted in Fluoromount aqueous mounting media, and analyzed by fluorescence microscopy (Zeiss). Fluorescence intensities were quantified by ImageJ and net mean fluorescence intensity calculated after subtraction of background fluorescence.

### 2.10 Characteristics of papillary tumors, tumor size, and histopathology scoring

To investigate the impact of alpha1‐oleate treatment on tumor size, all participants underwent outpatient cystoscopy at Visit 0. Tumors were re‐examined at Visit 7, prior to scheduled surgery. High‐quality photographs were obtained endoscopically using a flexible cystoscope (Olympus) before tumor removal by TURBT, following EAU Guideline recommendations.^21^ Changes in tumor size were assessed intra‐individually, by comparing paired images.

Multiple biopsies, obtained at the time of surgery, underwent histopathological evaluation using established criteria for grading and staging/invasiveness. Tissue sections were examined by a designated study uropathologist. Both grading systems (WHO 1973 and 2004/2016) were used for assessment. Biopsies from healthy tissue regions, located away from the tumor site, were also collected for comparative analysis.

### 2.11 RNA sequence analysis

RNA was isolated from biopsy specimens using the Illumina TruSeq Stranded mRNA Library Prep Kit (20020594), and libraries were multiplexed and sequenced on NextSeq 500/550 High Output Kits (v2.5 2 × 75 Cycles), generating an average of 22 million reads per sample. Raw sequencing data was demultiplexed using bcl2fastq (version 2.18), and abundance estimation was conducted using RSEM (1.3) with the human genome release 37/Ensemble 75.

Quality control measures were implemented, and data were visualized using dimensionality reduction techniques such as PCA, MA‐plots, and assessment of RNA‐seq intrinsic biases including GC bias, transcriptome complexity, and alignment quality. Differential expression analysis was carried out using R (version 3.4) and the limma and DESeq2 packages. Fold changes were determined by comparing tumors in the treated group to those in the placebo group. Genes with an absolute fold change >2.0 and *p* < 0.05 were considered differentially expressed. Heatmaps were generated using Gitools 2.1.1 software.

Ingenuity Pathway Analysis version 57,662,101 (IPA, Qiagen) software was used for the functional characterization of differentially expressed genes. Gene Set Enrichment Analysis (GSEA) was used to identify the cancer‐related biological processes and pathways affected by alpha1‐oleate treatment. Kyoto Encyclopedia of Genes and Genomes (KEGG) and Hallmark gene sets were used for enrichment analysis. Gene sets with *p* < 0.05 and false discovery rate (FDR) < 0.05 were considered statistically significant.

### 2.12 Statistical analysis

For assessing efficacy, the sample size was determined based on the analysis of changes in tumor cells from the placebo‐controlled part of the study.^19^ The normality of data distribution was assessed using the D'Agostino & Pearson normality test. Student's *t*‐tests were employed for data conforming to a Gaussian distribution, while the Mann–Whitney *U*‐test was used for non‐normally distributed datasets. Correlations were evaluated using Spearman correlation analysis. Kinetic data was analyzed utilizing the repeated measures 2‐way ANOVA test. Statistical analyses were conducted using Prism version 6.02 (GraphPad Software Inc.). *p* < 0.05 were considered statistically significant. All visual representations were produced by the study team.

## 3 RESULTS

Patients in the placebo‐controlled part of the study received intravesical instillations of alpha1‐oleate (1.7 mM, *n* = 20) or placebo (*n* = 20) on six occasions during 1 month preceding scheduled TURBT (CONSORT flow chart in Figure S1) and all patients completed the study. Patients in the subsequent dose‐finding part received a five times higher dose (8.5 mM, *n* = 6) or a 10‐times higher dose (17 mM, *n* = 3) of alpha1‐oleate. Primary end points were safety, the change in tumor size and tumor cell shedding into the urine. Secondary end points were evaluation of tumor biopsies by histopathology, quantification of alpha1‐oleate uptake by the tumor and tumor cells, apoptosis induction in the tumor and tumor cells and gene expression analysis by RNA sequencing of tissue samples obtained at TURB.

### 3.1 Safety

Severe, local or systemic drug‐related side effects were not detected in patients treated with 1.7, 8.5 or 17 mM of alpha1‐oleate (Grade III or IV). Systemic side effects were not observed, consistent with the known inactivation of the complex in serum. Local side effects related to the instillation procedure were observed in the treatment and placebo groups. These side effects were not more common in the treatment groups compared to the placebo group.^19^ Local side effects such as urgency and hematuria were recorded in 12/20 patients in the 1.7 mM group and 5/6 patients in the 8.5 mM group, compared to 11/20 in the placebo group (*p* > 0.05). All patients in the 1.7 and 8.5 mM groups completed the treatment. Two patients receiving 17 mM of alpha1‐oleate completed the study without drug related side effects. One patient receiving 17 mM of alpha1‐oleate experienced local side effects and dropped out of the study after visit 3.

All urine samples from the included patients were analyzed resulting in a sample size of 2 × 6 × 6 (*n* = 72) for the 8.5 mM group, 2 × 6 × 20 (*n* = 240) for the 1.7 mM group and 2 × 6 × 20 samples for the placebo group (*n* = 240). The number of tumors was evaluated before and after treatment by examination of the video recordings at cystoscopy. The final numbers are 36 for the 1.7 mM, 25 for the 8.5 mM and 40 for the placebo group. There were 14 tumors left to biopsy after treatment in the 8.5 mM treatment group explaining the number of samples for mRNA and immunohistochemistry.

### 3.2 Effects on tumor number, size, and appearance

#### 3.2.1 Changes in individual tumors recorded by endoscopy

**FIGURE 1:**
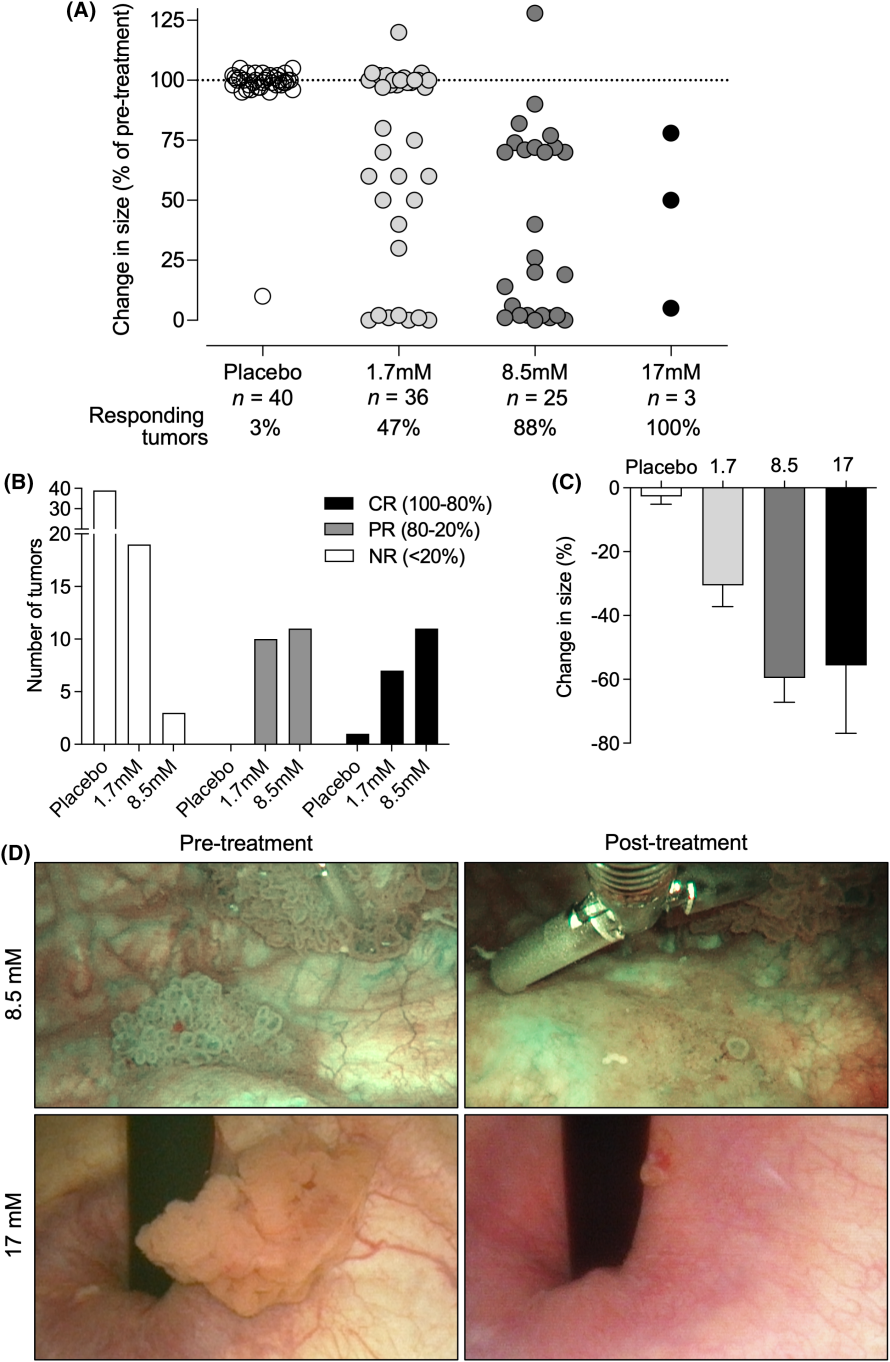
Dose‐dependent effects of alpha1‐oleate on tumor size after intravesical instillation. (A) Changes in tumor size were evaluated by cystoscopy, comparing pre‐treatment to post‐treatment images. The change in tumor size was calculated in percent of the pre‐treatment size, using ImageJ to quantify for remaining tumors. A loss of tumor was recorded as 0%. A significant, dose‐dependent reduction in tumor size was detected. Individual tumor sizes are shown. (B) Number of complete responding (CR, 100% to 80% reduction in size) tumors, partial responding (PR, 80% to 20% reduction in size), and non‐responding (NR, <20% reduction in size) tumors, after treatment. Fisher's exact test compared to Placebo, *p* < 0.001 for 1.7 mM and *p* < 0.001 for 8.5 mM. (C) Mean reduction in size for all evaluated tumors. Mean ± SEM, Kruskal–Wallis test using Dunn's multiple comparison compared to Placebo, *p* = 0.007 for 1.7 mM, *p* < 0.001 for 8.5 mM and *p* = 0.05 for 17 mM. (D) Examples of images captured during cystoscopy, comparing the tumor sizes before and after alpha1‐oleate treatment.

The tumor number and tumor size were evaluated before and after treatment by examination of video recordings performed at cystoscopy. Significant, dose dependent changes in tumor number and tumor size were detected by recording the same tumor area at the time of diagnosis and post treatment, before transurethral resection (Figure 1A). Tumor sizes were quantified using ImageJ software, using the tip of the clamp as a scale reference. The tumor response was classified as complete (80%–100% reduction), partial (20%–80% reduction) or stable disease (<20% change). Progression was defined as an increase in tumor size (>100%). In addition to the main tumor, quantification included additional tumors adjacent to the main tumor, captured in the same field of view.

A complete or partial response was observed in 88% of the tumors in the 8.5 mM group, compared to 47% in the 1.7 mM group (Figure 1B). The frequency of tumors with a complete response increased from 27% in the 1.7 mM to 44% in the 8.5 mM group. The average size reduction was 59% in the 8.5 mM group compared to 30% in the 1.7 mM group and 5% in the placebo group (Figure 1C).

The two patients receiving 17 mM of alpha1‐oleate showed a strong response in 3/3 tumors, with one complete response of a larger tumor and partial responses in two tumors in the second patient. By visual inspection the bladder surfaces in the treated patients appeared paler and less vascularized than in the placebo group. Remaining tumors further showed a change in surface characteristics, with tissue stroma clearly visible at the surface, supporting a loss of tumor cells and tumor mass after compared to before treatment (Figure 1D).

#### 3.2.2 Cell shedding

**FIGURE 2:**
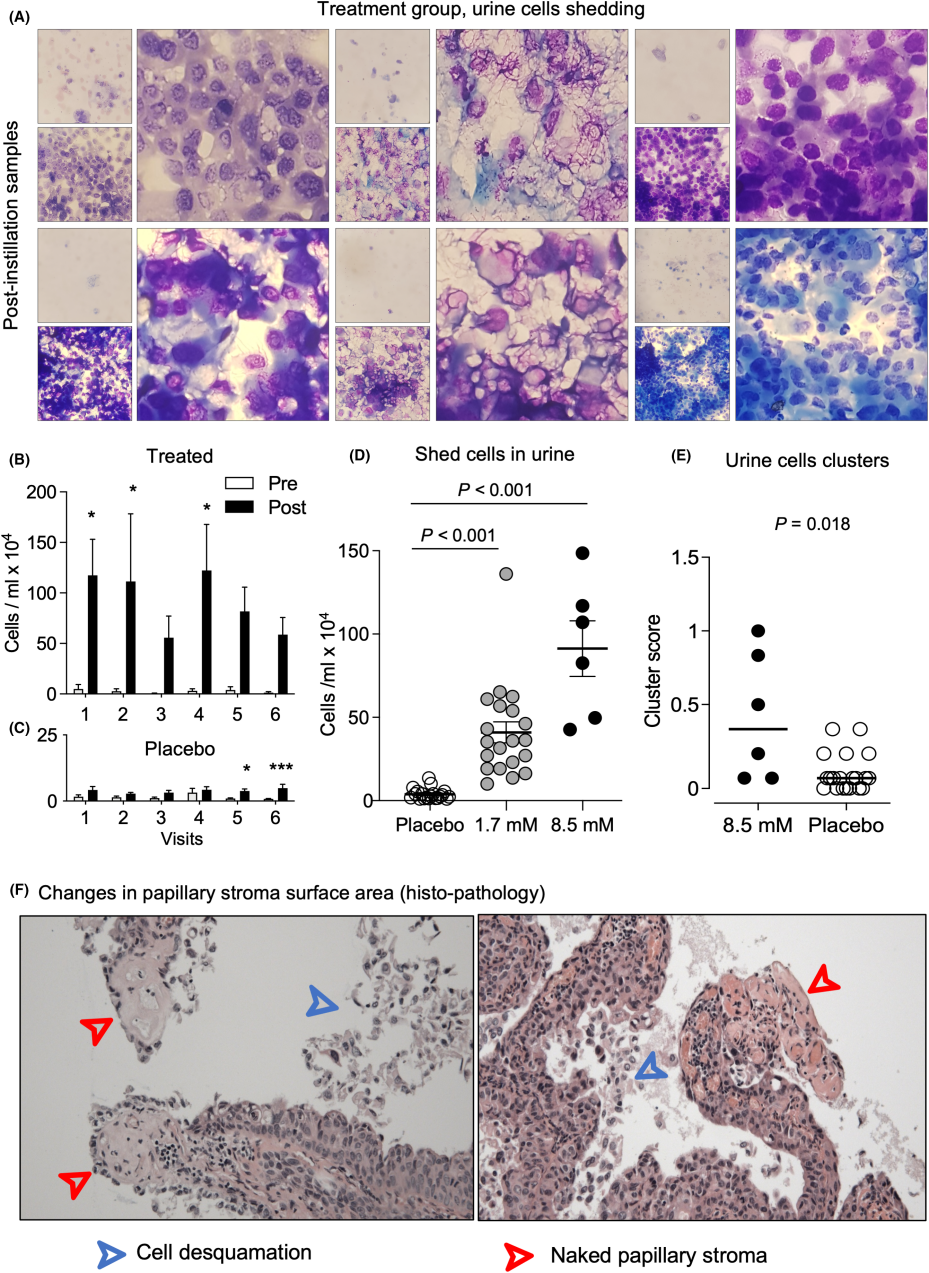
Dose‐dependent cell shedding into the urine in response to intravesical alpha1‐oleate instillations. Cell shedding was quantified at each visit, before and approximately 2 h after the instillation of 1.7 or 8.5 mM of alpha1‐oleate, or placebo. (A) Representative images illustrating the increase in cell shedding in post instillation samples in different patients treated with 8.5 mM of alpha1‐oleate. (B) Cell numbers in urine samples obtained pre (White) and post (Black) alpha1‐oleate instillation. Mean ± SEM of six patients per visit, multiple comparisons with 2‐way ANOVA using Sidak's correction, *p* = 0.04 for visit 1, *p* = 0.05 for visit 2, *p* = 0.02 for visit 4, and *p* > 0.2 for visits 3, 5, 6, * *p* < 0.05, ** *p* < 0.01, *** *p* < 0.001. (C) Cell numbers in the placebo group. Urine samples were obtained pre (White) and post (Black) PBS instillation. Mean ± SEM of 20 patients per visit, multiple comparisons with 2‐way ANOVA using Sidak's correction, *p* = 0.5 for visit 1, *p* > 0.2 for visits 2, 3, 4, *p* = 0.02 for visit 5 and *p* < 0.001 for visit 6. (D) Scatter plot of individual patient response (mean of 6 visits, post‐instillation samples), comparing the number of cells shed in alpha1‐oleate treated patients versus placebo. Mean ± SEM, Kruskal–Wallis test. (E) Scatter plot of individual patient response (mean of 6 visits per patient), comparing cluster scoring of shed urine cells in 8.5 mM alpha1‐oleate treated patients versus placebo. Medians are indicated by the horizontal bar, Kruskal–Wallis test. (F) Histopathology of 8.5 mM alpha1‐oleate treated tumor biopsies, indicating tumor fragmentation and cell desquamation, and resulting changes in the tumor surface with denuded papilli and exposure of the papillary stroma.

Intravesical alpha1‐oleate instillations were followed by rapid, dose‐dependent tumor cell shedding, resulting in increased cell numbers in the urine after each instillation, compared to each pre‐instillation sample (Figure 2A,B and Figure S2). All urine samples from the included patients were analyzed resulting in a sample size of 2 × 6 × 6 (*n* = 72) for the 8.5 mM group, 2 × 6 × 20 (*n* = 240) for the 1.7 mM group and 2 × 6 × 20 samples for the placebo group (*n* = 240). The response was dose dependent, as defined by significantly higher cell numbers in patients receiving 8.5 mM compared to 1.7 mM of alpha1‐oleate. Significant cell shedding was not detected in the placebo group after instillations of PBS (Figure 2C–E). The tumor origin of the shed cells was more clearly documented in the 8.5 mM group, compared to the 1.7 mM group, further supporting the dose‐dependent anti‐tumor effect.

#### 3.2.3 Tumor histopathology

Superficial cell desquamation and tumor fragmentation were also detected by histopathology of biopsy specimens obtained at surgery (Figure 2F and Figure S3). Desquamation was defined, as the presence of single cells or larger cell clusters, without connection to the stroma. Tumor fragmentation and shedding left naked papillary stroma, defined by a loss of papillary structure and exposed, denuded basement membranes. Regressive changes were present in most of the tumors, defined by a loss of cellular structure, in large areas, as well as hyalin deposits.

#### 3.2.4 Alpha1‐oleate uptake by tumor cells and tissues

**FIGURE 3:**
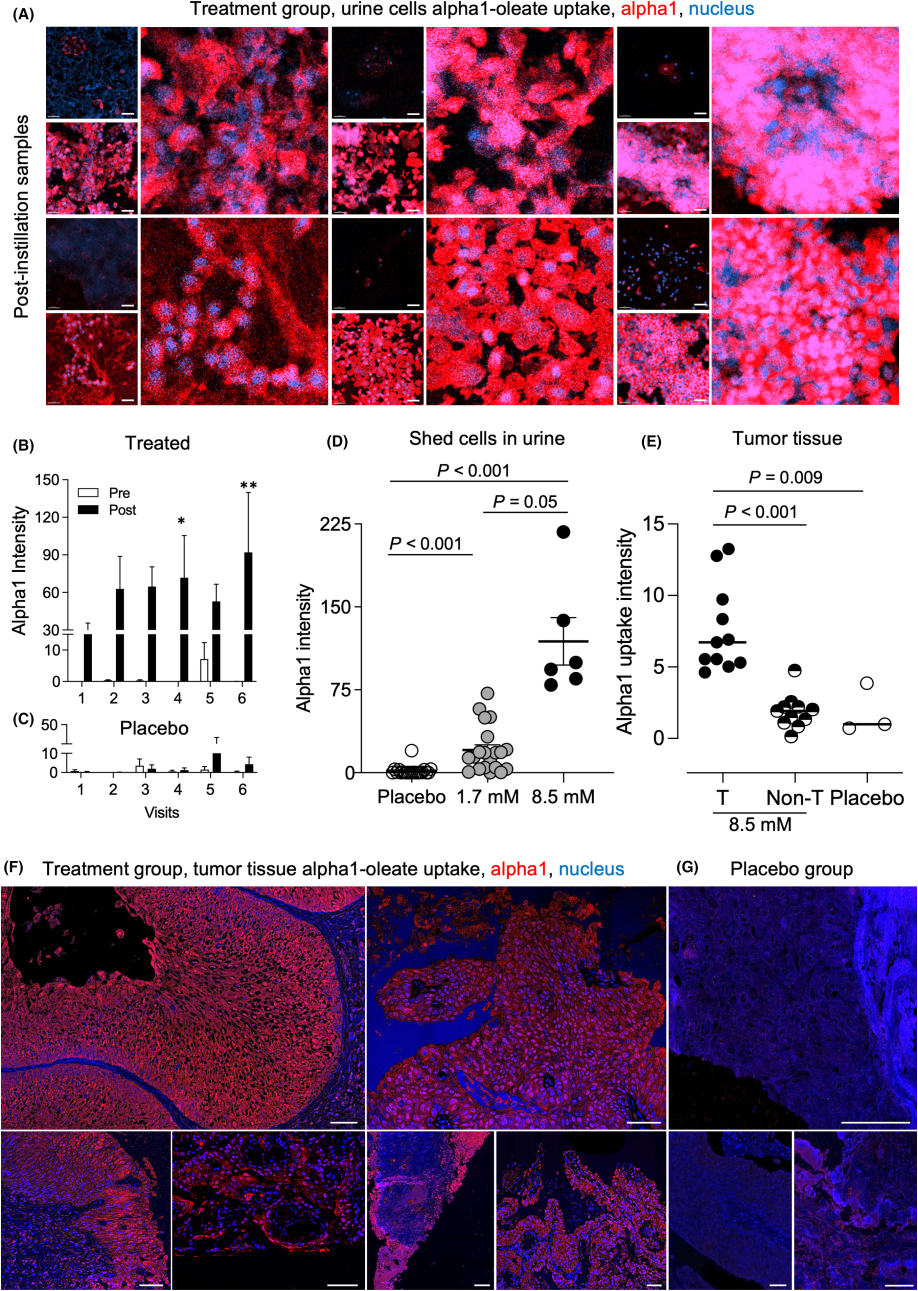
Alpha1‐oleate uptake by tumor cells and tumor tissue. Alpha1 uptake was quantified in shed cells and tumor biopsies using polyclonal anti‐alpha1 antibodies. Fluorescence intensity was quantified, and arbitrary units were calculated after subtraction of background staining in the pre‐inoculation sample, visit 1. (A) Representative images of the alpha1 content of shed cells pre‐ and post‐ instillations (Red = alpha1, Blue = nucleus). (B) Increase in alpha1 content in shed cells, comparing pre (white) and post (Black) treatment samples from each visit. Mean ± SEM of six patients per visit, multiple comparisons with 2‐way ANOVA using Sidak's correction, *p* = 0.04 for visit 4, *p* = 0.005 for visit 6, and *p* > 0.08 for other visits. * *p* < 0.05, ** *p* < 0.01, *** *p* < 0.001. (C) Lack of alpha1 staining in the placebo group, pre (white) and post (Black) inoculation of PBS. Mean ± SEM of 20 patients per visit, multiple comparisons with 2‐way ANOVA using Sidak's correction, *p* > 0.99 for all visits. (D) Scatter plot of individual patient response (mean of 6 visits, post‐instillation samples), demonstrating dose‐dependent increase in alpha1 uptake comparing 8.5 and 1.7 mM of alpha1‐oleate and placebo. Mean ± SEM, Kruskal–Wallis test, *p* < 0.001 for both treatments compared to placebo. (E) Alpha1 staining intensity of tissue biopsies from patients treated with alpha1‐oleate and placebo. Scatter plot of individual tumors demonstrating an increase in alpha1 content in tumor regions compared to non‐tumor regions. Median are indicated by the horizontal bar, Kruskal–Wallis test with Dunn's multiple comparisons. (F) Representative images of alpha1 staining in tumor tissue from individual patients treated with alpha1‐oleate (8.5 mM), (Red = Alpha1, Blue = nucleus). (G) Representative images of alpha1 staining in tumor tissue from individual patients receiving placebo. Scale bar 40 μm (A), 50 μm (F, G).

Alpha1‐oleate was detected by immunohistochemistry inside shed tumor cells and in tumor tissue. Staining was detected in post‐instillation samples from all alpha1‐oleate treated patients, illustrating the efficiency of uptake by the tumor. Cells in urine showed a rapid, dose‐dependent increase in alpha1‐oleate content at all visits post instillation, compared to the pre‐instillation samples (Figure 3A–D and Figure S4). The uptake of alpha1‐oleate was further quantified by staining of tissue biopsies obtained at TURBT (Figure 3E,G). Strong alpha1‐oleate staining in tumor areas was detected in patients receiving 8.5 mM of alpha1‐oleate and was significantly more pronounced in tumor areas than in healthy tissue areas of the same specimen, confirming the preferential uptake by tumor tissue (Figure 3E,F and Figures S5, S6). Alpha1‐oleate staining was not observed in tissue sections from the placebo group (Figure 3E,G).

#### 3.2.5 Apoptosis induction

**FIGURE 4:**
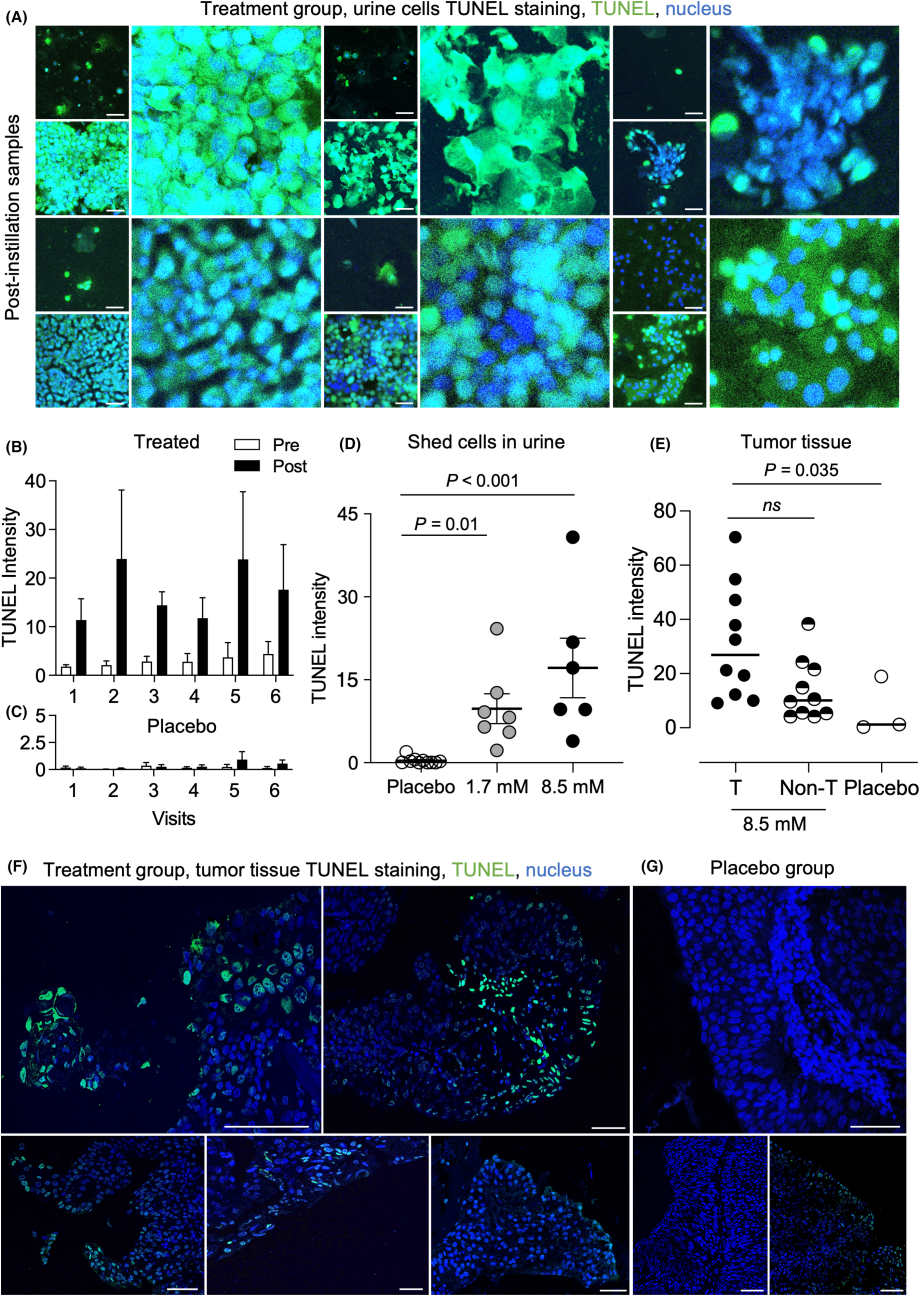
DNA strand breaks in tumor cells and tumor tissue. TUNEL staining in shed cells and tumor tissue of patients treated with alpha1‐oleate (8.5 mM). Fluorescence intensity was quantified, and arbitrary units were calculated after subtracting the background staining in TUNEL negative healthy tissues from each patient. (A) Representative images of TUNEL staining in cells pre‐ and post‐ instillations (Green = TUNEL, Blue = nucleus). (B) Increase in TUNEL staining intensity in shed cells, comparing pre (White) and post (Black) treatment samples for each visit. Mean ± SEM of six patients per visit, multiple comparisons with 2‐way ANOVA using Sidak's correction, *p* = 0.08 for visit 2, *p* = 0.13 for visit 3, *p* > 0.5 for other visits. (C) Low TUNEL signal in the placebo group, pre (White) and post (Black) inoculation of PBS. Mean ± SEM of 20 patients per visits, multiple comparisons with mixed effect analysis using Sidak's correction, *p* > 0.6 for all visits. (D) Scatter plot of individual patient response (mean of 6 visits, post‐instillation samples), demonstrating increase in dose dependent TUNEL intensity comparing the 8.5, 1.7 mM and placebo groups. Mean ± SEM, Kruskal–Wallis test, *p* < 0.01 for both treatment. (E) TUNEL staining intensity in tumor tissue from patients treated with alpha1‐oleate compared to placebo. Scatter plot of individual tumors demonstrating higher TUNEL staining in alpha1‐oleate treated tumor regions compared to non‐tumor regions. Median, Kruskal–Wallis test with Dunn's multiple comparisons. (F) Representative images of TUNEL staining in tumor tissue from individual patients treated with alpha1‐oleate (8.5 mM), (Green = TUNEL, Blue = nucleus). (G) Representative images of TUNEL staining in tumor tissue from individual patients receiving placebo. Scale bar 40 μm (A), 50 μm (F,G).

Apoptosis was quantified by TUNEL staining, which detects double strand DNA breaks. Cells shed post alpha1‐oleate instillation were compared to the pre‐instillation samples (Figure 4A and Figure S7). A marked increase in TUNEL staining was detected post‐instillation, compared to the placebo group, with a dose‐dependent increase (Figure 4B–D). TUNEL staining was further quantified in tissue biopsies obtained at TURBT. Peripheral TUNEL staining was detected in 9/10 stained tumors, but the staining intensity was lower than in the shed cells, suggesting that cells from the TUNEL positive tumor areas detach into the urine (Figure 4E–G and Figure S8).

### 3.3 Gene expression analysis

**FIGURE 5:**
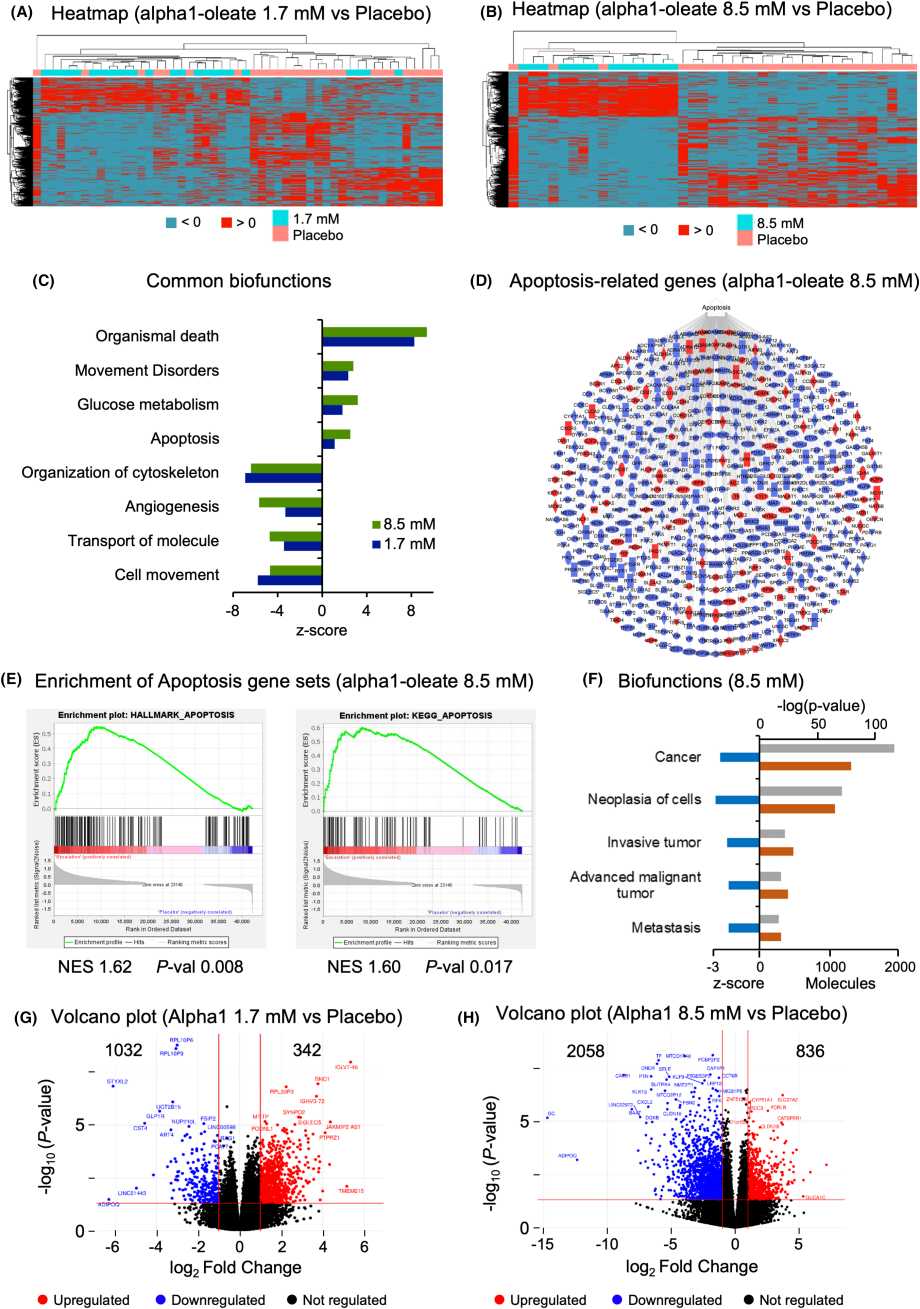
Gene expression in tumors from patients receiving 1.7 and 8.5 mM of alpha1‐oleate, compared to placebo. Tissue RNA was extracted from biopsies obtained at TURBT, post treatment with alpha1‐oleate or from the placebo group. (A,B) Heatmap clustering of individual tumors, based on genes differentially expressed between 1.7 mM‐treated or 8.5 mM treated and placebo tumors (cut‐off FC >2, *p* < 0.05, compared to placebo tumors, Red = upregulated, Blue = downregulated). The clustering of the 8.5 mM treated group was more pronounced than the 1.7 mM‐treated group. (C) Functional classification of genes regulated in the 1.7 and 8.5 mM treatment groups, top common biofunctions are shown. Apoptosis was activated and cancer‐related functions inhibited (angiogenesis, tumor cell movement) (z‐score >2, *p* < 0.05). (D) Network of apoptosis related genes in the 8.5 mM treatment group. (E) GSEA analysis identifying apoptosis related gene sets as significantly enriched in treated tumors. (F) Top cancer associated functions in 8.5 mM treated tumors. Cancer, neoplasia, invasion, metastasis and advance malignant tumor functions were strongly inhibited (z‐score < −2, *p* < 0.05). (G) Volcano plot of genes regulated in response to alpha1‐oleate treatment in the 1.7 mM treatment group. One thousand and thirty‐two genes were inhibited and 342 were activated compared to placebo tumors. (H) Volcano plot of genes regulated in response to alpha1‐oleate treatment in the 8.5 mM treatment group. Two thousand and fifty‐eight genes were inhibited and 836 genes were activated compared to placebo tumors.

The tumor response to alpha1‐oleate or placebo was characterized in greater detail by RNA sequencing. RNA was extracted from tumor tissue obtained at TURBT, and gene expression was compared between patients receiving alpha1‐oleate treatment or placebo. A pronounced, dose‐dependent effect on gene expression was detected by clustering analysis (Figure 5A,B), consistent with the clinical effects.

The induction of apoptosis was confirmed by gene expression analysis, as the activation of genes involved in programmed cell death in the 1.7 and 8.5 mM treatment groups, compared to placebo (z‐score >2, *p* < 0.05) (Figure 5C). Genes associated with apoptosis were differentially expressed, comprising about 20% of all regulated genes in both treatment groups. Apoptosis genes were more strongly regulated in the 8.5 mM treatment group (510 genes) than in the 1.7 mM treatment group (273 genes), with a combined pattern of activation and inhibition (Figure 5D and Figure S9). GSEA analysis identified gene sets associated with classical apoptosis as well as the KEGG pathway of apoptosis, as enriched in the 8.5 mM treatment group compared to placebo (Figure 5E). Significantly enriched genes included Caspases 2, 8, 9, *Bcl2*, *RhoB*, *Ras*, *TNFR*, *RelA*, and *p53* (Figure S10).

The overall inhibition of gene expression in treated tissues is illustrated in the Volcano plots (Figure 5G,H). Cancer‐related biofunctions were inhibited, including angiogenesis, tumor cell migration and tumor invasion (Figure 5F), with additional effects on cancer related genes, categorized as neoplasia (530 genes), tumor invasion, TGFB genes (38 genes), connective tissue deterioration and inflammation. Cancer‐related biofunctions were more strongly inhibited in the 8.5 mM group, suggesting that alpha1‐oleate treatment broadly inhibits critical cancer functions, in a dose‐dependent manner (Figure 5F and Figure S9).

**FIGURE 6:**
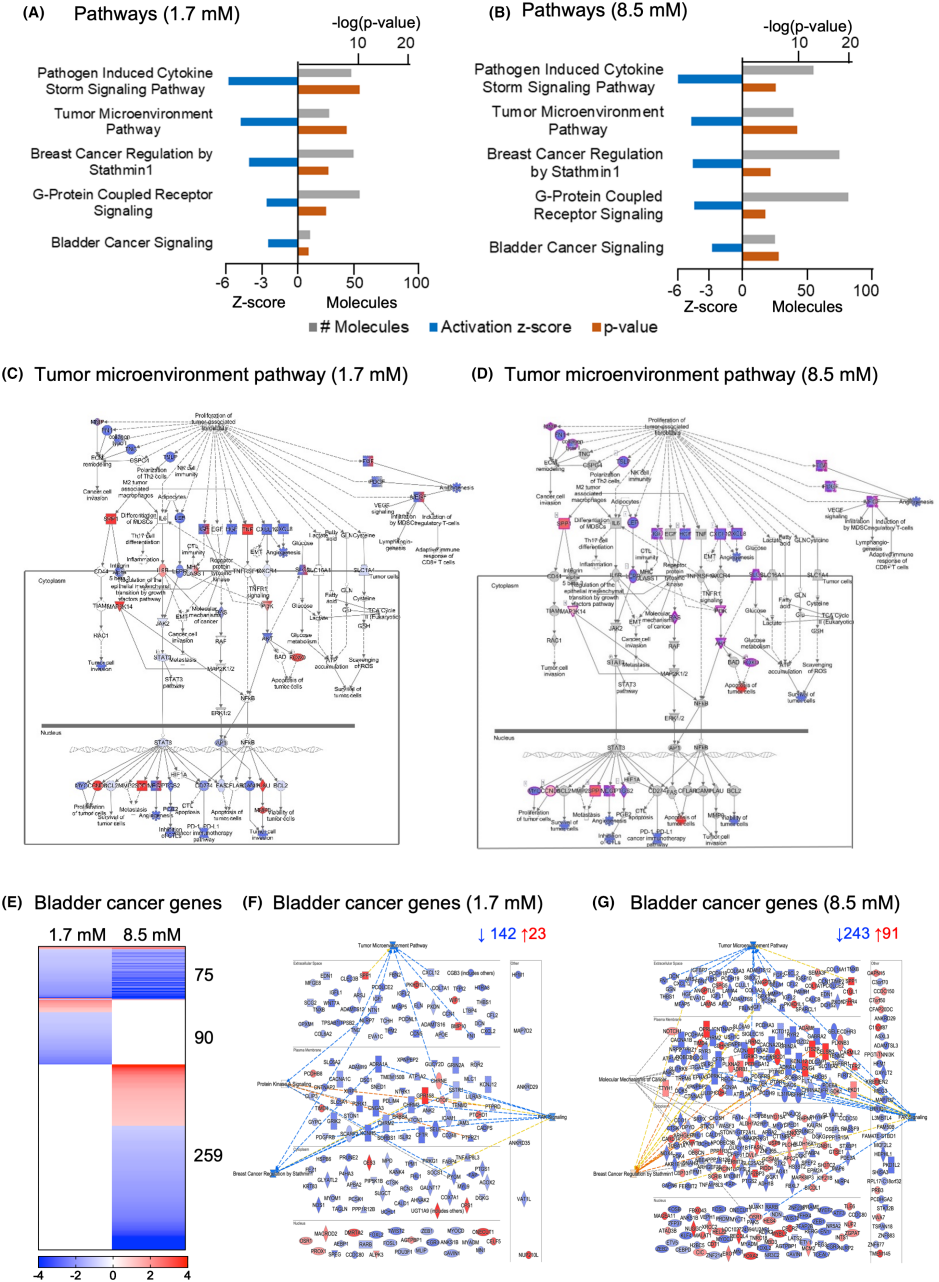
Inhibition of cancer gene expression and bladder cancer related genes in treated tumors. (A) Top inhibited pathways in 1.7 mM treated tumors (Blue = z‐score, Orange = *p*‐value and Gray = molecules). (B) Top inhibited pathways in 8.5 mM treated tumors (Blue = z‐score, Orange = *p*‐value and Gray = molecules). Both treatment groups showed strong inhibition of Cytokine Storm Signaling, Tumor Microenvironment genes, Breast Cancer Regulation by Stathmin1 and Bladder Cancer Signaling pathways (z‐score < −2, *p* < 0.05). (C, D) Regulation of Tumor Microenvironment Pathway genes in 1.7 or 8.5 mM treated tumors (Blue = inhibition, Red = activation). (E) Heatmap comparing bladder cancer‐related gene expression in 1.7 and 8.5 mM treated tumors. (F, G) Network of bladder cancer related genes in 1.7 or 8.5 mM treated tumors. Genes related to the tumor microenvironment, focal adhesion signaling, breast cancer regulation by stathmin were differentially expressed compared to placebo, as well as Protein Kinase A Signaling in 1.7 mM treated tumors, and molecular mechanisms of cancer in the 8.5 mM treated tumors.

Pathway analysis further confirmed the dose‐dependent inhibition of the tumor microenvironment pathway in both treatment groups, as well as the bladder cancer‐signaling pathway (Figure 6A,B). Down‐regulated genes in the tumor microenvironment pathway included fibroblast growth factors and vascular endothelial growth factors (*FGF1*, *FGF5*, *FGF7*, *FGF10*, and *VEGFD*), regulating angiogenesis, transforming growth factor beta genes (*TGFB2* and *TGFB3*), regulating epithelial mesenchymal transition and cytotoxic T lymphocytes and Insulin growth factor 1 (*IGF1*), regulating cancer cell invasion and metastasis (Figure 6C,D). Additionally, the innate immune response was strongly inhibited, with a broad dose‐dependent anti‐inflammatory effect defined as a reduction in cytokine storm signaling as well as G protein‐coupled receptor signaling (Figure 6A,B). Chemokine and cytokine expression was inhibited in both treatment groups, predicting effects on the innate and adaptive immune responses. This included *CCL2*, *CCL11*, *CCL20*, *CXCL1*, *CXCL2*, *CXCL12*, *IL1B*, and *IL17D*.

#### 3.3.1 Bladder cancer genes

A large number of genes related to bladder cancer were affected by alpha1‐oleate treatment compared to the placebo group. Genes previously reported to be abundantly expressed in different bladder cancer subtypes were inhibited in both groups, with a shared pattern of inhibition (*n* = 75 shared genes, Figure 6E–G). Functional analysis identified strongly down‐regulated genes affecting cancer cell growth (Figure 6F,G), including the growth factor receptor *PDGFRB*, early growth response 3 (*EGR3*), a VEGF dependent angiogenesis biomarker, and protein kinase genes (*PRKG1*).^22^, ^23^, ^24^, ^25^ Transcription factors were inhibited in both treatment groups, potentially effecting tumor development and metastasis (*TWIST2*, *FOSL1*, and *ZEB1*, Figure 6F,G).^26^, ^27^

Additional genes, associated with bladder cancer were inhibited in the 8.5 mM treatment group, including the transcriptional repressor *PRAME*, which inhibits apoptosis (FC = −82), Transferrin (*TF*), which stimulates cell proliferation (FC = −64) and the ATP Binding Cassette Subfamily B Member 5 (*ABCB5*) (FC = −47). Top upregulated genes included the complement factor related gene (*CFHR4*), the transcriptional regulator (*FOXA2*), the urotensin‐2 receptor (*UTS2R*) and programmed cell death protein 1, PD‐1 (*PDCD1*), which is a target for checkpoint inhibition therapy. In addition, Ca^2+^ voltage‐gated channels, and ATP transporters were upregulated, consistent with the membrane effects of alpha1‐oleate, especially in tumor cells.^28^

Pronounced effects on gene expression and changes in the tumor environment were detected in all patients treated with 8.5 mM of alpha1‐oleate, suggesting a general response characterized by the activation of apoptosis and inhibition of cancer related gene networks in tumor tissues.

## 4 DISCUSSION

Alpha1‐oleate targets tumors and affects them by several mechanisms of action, highlighted by the results of this study. First, the complex acts directly on bladder tumors, killing cancer cells by an apoptosis like mechanism and inducing rapid detachment of tumor cells and tumor fragments, into the urine. Second, alpha1‐oleate inhibits gene expression, reprogramming the tumor environment by inhibiting classical oncogenes and cancer related functions. Third, alpha1‐oleate triggers a rapid and broad immune response, with anti‐tumor characteristics. The reduction in tumor number and tumor size by alpha1‐oleate treatment and the lack of severe side effects confirmed in this study, identify alpha1‐oleate an innovative approach to the treatment of bladder cancer, with a promising potential. This multifaceted mechanism of action and tumor selectivity distinguishes alpha1‐oleate from current immunotherapies and chemotherapies currently used in bladder cancer.

This study identified potent, dose‐dependent anti‐tumor effects of alpha1‐oleate in patients with NMIBC. Following intravesical instillation, alpha1‐oleate was taken up by tumor tissue, triggering rapid cell shedding and the induction of apoptosis, resulting in a loss of papillary structure and a reduction in tumor size. Alpha1‐oleate treatment further affected gene expression inhibiting cancer‐related gene networks and activating programmed cell death. Inhibited functions included, tumor cell growth, tumor invasion, angiogenesis, and metastasis as well as molecules previously associated with bladder cancer and treatment outcome. Visual inspection of the tumor area, documented by video recording, added precision to the tumor analysis. The observations made illustrate the complexity of the bladder cancer disease and challenges of defining treatment effects. The effects on the main tumor were accompanied by broader changes in the tissues surrounding this tumor. Effects on smaller satellite tumors were observed and tissue vascularization was reduced, consistent with the inhibition of genes involved in neo‐angiogenesis. The results support a combined treatment effect resulting in the removal of tumors or reduction in tumor size, as well as the loss of typical cancer characteristics in the remaining tumor tissues.

Alpha1‐oleate is derived from the HAMLET complex, which kills a range of tumor cells in vitro and has shown therapeutic efficacy in cancer models with a high degree of selectivity for tumor tissue.^12^, ^13^, ^14^, ^15^, ^16^, ^17^, ^29^ HAMLET is formed by the globular 14.2 kDa protein alpha‐lactalbumin, which binds to oleic acid, the naturally occurring lipid cofactor in HAMLET. The N‐terminal, 39‐residue alpha‐helical peptide of human alpha‐lactalbumin forms the oleic acid complex, alpha1‐oleate, which reproduces tumoricidal effects of HAMLET. In alpha1‐oleate, the alpha1 peptide retains significant alpha‐helical structure, as shown by circular dichroism (CD) spectra, NMR spectroscopy and molecular modeling.^19^

Early studies showed that the sensitivity to HAMLET is guided by criteria generally accepted as “Hallmarks” of cancer.^30^, ^31^ Classical oncogenes like MYC, RAS and HIF1α were shown to determine HAMLET sensitivity, using a short hairpin RNA screen.^30^ Similarly, oncogene signaling pathways were inhibited in alpha1‐oleate treated tumor cells and the expression of cancer related genes was markedly reduced.^17^, ^19^, ^32^ This study defines potent, dose‐dependent effects on cancer‐related gene expression pathways in treated patients. The tumor microenvironment pathway was strongly inhibited, affecting Ras, PI3K‐AKT signaling, tumor angiogenesis, and tumor cell survival via downregulation of VEGF signaling. Gene expression analysis further revealed effects of alpha1‐oleate on bladder cancer related genes, which were strongly inhibited. This included TF/TfR, which is associated with drug resistance in multiple cancers^33^, ^34^ and *PRAME*, which is overexpressed in NMIBC tumors that respond poorly to chemotherapy.^35^ The results suggest that alpha1‐oleate reprograms gene expression, moving the bladder tumors away from the cancer‐related gene expression profile, towards a healthy phenotype.

Control of tumor cell death by induction of apoptosis has obvious advantages. Selective removal tumor tissue by apoptosis would be useful to minimize effects on healthy tissue and the properties of alpha1‐oleate are interesting from that perspective. In this study alpha1‐oleate treatment was shown to trigger a rapid dose‐dependent apoptosis‐like response, visible by TUNEL staining of cells harvested about 2 h after intravesical instillation. About 20% of regulated genes in tumor tissue were apoptosis related, confirming the effect of treatment compared to placebo. The broad activation of apoptosis related genes in remaining tumor tissue suggesting that the response continues after the end of treatment. Additional cell death mechanisms may be activated by alpha1‐oleate, including autophagy, but inhibitors of well‐established cell death pathways never completely abolish the effects of alpha1‐oleate, consistent with the broad effect on genes involved in organismal death, a category of genes related to survival.

The response of bladder cancers to Bacillus Calmette–Guérin (BCG) therapy has been extensively investigated, using gene expression analysis, DNA and RNA sequencing technology.^36^, ^37^, ^38^ Gene expression profiles have been associated with outcomes such as BCG resistance of the tumor or recurrence rates. Sanders et al. defined genomic profiles in high grade T1 NMIBC tumors with a durable response to BCG and several inflammatory pathways were detected among the responders.^36^ *MCL1* was identified as a candidate for further study and potential biomarker. Kim et al. examined the predictability of BCG resistance using an extensive data set of tumors from 80 patients and identified gene signatures associated with recurrence‐ or progression‐free survival^37^ and the gene profiles identified in tumor tissue before BCG treatment were the only independent predictors of recurrence or progression in the data set. The response to BCG therapy included strong inhibition of tumor microenvironment genes and immunomodulators including *TNFA*, *IL17*, and *IL6*, suggesting similarities in gene expression profiles between patients undergoing BCG or alpha1‐oleate therapy.^39^ Interestingly, a recent proteomic screen detected a significant immune response to alpha1‐oleate in treated patients and identified a highly significant overlap with the immune response reported in BCG treated patients.^20^ This significant effect of alpha1‐oleate on cytokine secretion further confirms the effects on gene expression, at the proteomic level, adding a further mechanism to the protective potential alpha1‐oleate as well as its minimal toxicity.

The current first line of NMIBC treatment is TURBT, followed by intravesical chemotherapy or immunotherapy, using mitomycin C or BCG instillations. Recurrence rates are high and inadequate supply of immunotherapy and chemotherapy worldwide limits the treatment options. Valrubicin and pembrolizumab have been approved for treatment of BCG resistant disease,^40^ but reports suggest poor long‐term survival,^41^ emphasizing the complexity of bladder cancer therapy. The recently approved Nadofaragene firadenovec (also known as rAd‐IFNa/Syn3) based on delivery of human interferon alfa‐2b cDNA into the bladder by a replication‐deficient recombinant adenovirus, has shown efficacy for BCG‐unresponsive NMIBC.^42^ The present study defines a promising response to alpha1‐oleate treatment of newly diagnosed NMIBC or early recurrences and provides insights into the cellular and molecular basis for these promising effects. The reduction in tumor number and tumor size by alpha1‐oleate treatment and the lack of severe side effects confirmed in this study, identify alpha1‐oleate as an innovative approach to the treatment of bladder cancer, with a promising potential in patients with NMIBC, initially as a neoadjuvant, targeting newly diagnosed tumors or recurrences.

## AUTHOR CONTRIBUTIONS

**Farhan Haq:** Data curation (equal); formal analysis (equal); methodology (equal); writing – review and editing (equal). **Samudra Sabari:** Data curation (equal); formal analysis (equal); methodology (equal); writing – review and editing (equal). **Jaromir Háček:** Data curation (equal); investigation (equal); methodology (equal). **Antonín Brisuda:** Data curation (equal); investigation (equal); methodology (equal). **Ines Ambite:** Formal analysis (equal); methodology (equal); visualization (equal); writing – review and editing (equal). **Michele Cavalera:** Data curation (equal); methodology (equal). **Parisa Esmaeili:** Data curation (equal). **Murphy Lam Yim Wan:** Data curation (equal). **Shahram Ahmadi:** Data curation (equal). **Marek Babjuk:** Conceptualization (equal); formal analysis (equal); investigation (equal); writing – review and editing (equal). **Catharina Svanborg:** Conceptualization (equal); formal analysis (equal); methodology (equal); visualization (equal); writing – original draft (equal); writing – review and editing (equal).

## FUNDING INFORMATION

This research is funded by the Swedish Cancer Society (Cancerfonden), the Swedish Research Council, the Royal Physiographic Society in Lund, HAMLET BioPharma AB, Lund, Sweden and the European Union's Horizon 2020 research and innovation program under grant agreement No. 954360.

## CONFLICT OF INTEREST STATEMENT

C.S., I.A. and M.L.Y.W. hold shares in Hamlet BioPharma, as representatives of scientists in the HAMLET group. Patents protecting the use of alpha1‐oleate have been filed. No specific patents have been filed based on this study.

## ETHICS STATEMENT

The study was approved by the State Institute for Drug Control (SUKL) in the Czech Republic; number 273799/17‐I and the Ethics Committee of the Motol University Hospital; number EK‐786/17. Informed Consent Statement: Informed consent was obtained from all subjects involved in the study. Clinical Trail Registry Statement: This dose escalation part was an extension of the single center, placebo controlled, double blinded randomized Phase I/II interventional clinical trial of NMIBC (EudraCT Number: 2016–004269‐14 and ClinicalTrials.gov NCT03560479). Animal Studies Statement: N/A.

## Supporting information


Data S1:

## Data Availability

Data supporting findings of this study are available in the paper and supplementary data files. The RNA sequencing data generated in this study have been deposited in the Gene Expression Omnibus (GEO) database under accession number GSE172112 and the BioProject database under accession number PRJNA1119936.
