# Supplementary material for: Clinical and molecular response to alpha1‐oleate treatment in patients with bladder cancer
Source: Cancer Med. 2024 Sep 10;13(17):e70149. doi: 10.1002/cam4.70149 (PMC11386334; doi:10.1002/cam4.70149)
Supplement: Supplementary file 1 — Data S1: [file CAM4-13-e70149-s001.pdf]

Supplementary Material

Clinical and molecular response to alpha1-oleate treatment in patients with bladder cancer

Farhan Haq[1#], Samudra Sabari[1#], Jaromir Háček[2], Antonín Brisuda[3], Ines Ambite[1], Michele Cavalera[1], Parisa Esmaeili[1], Murphy Lam Yim Wan[1], Shahram Ahmadi[1], Marek Babjuk[3] and Catharina Svanborg[1*]

1. Division of Microbiology, Immunology and Glycobiology, Department of Laboratory Medicine, Faculty of Medicine, Lund University, Sweden
2. Department of Pathology and Molecular Medicine, Motol University Hospital, 2nd Faculty of Medicine, Charles University Praha, Prague, Czech Republic.
3. Department of Urology, Motol University Hospital, 2nd Faculty of Medicine, Charles University Praha, Prague, Czech Republic.

[#] These authors contributed equally

*** Corresponding author:**
Catharina Svanborg, Department of Microbiology, Immunology and Glycobiology, Lund University, Institute of Laboratory Medicine, Klinikgatan 28, BMC B13, 222 42 Lund, Sweden. catharina.svanborg@med.lu.se, +46 46 222 70 44.

Supplementary Figure 1

CONSORT flow chart

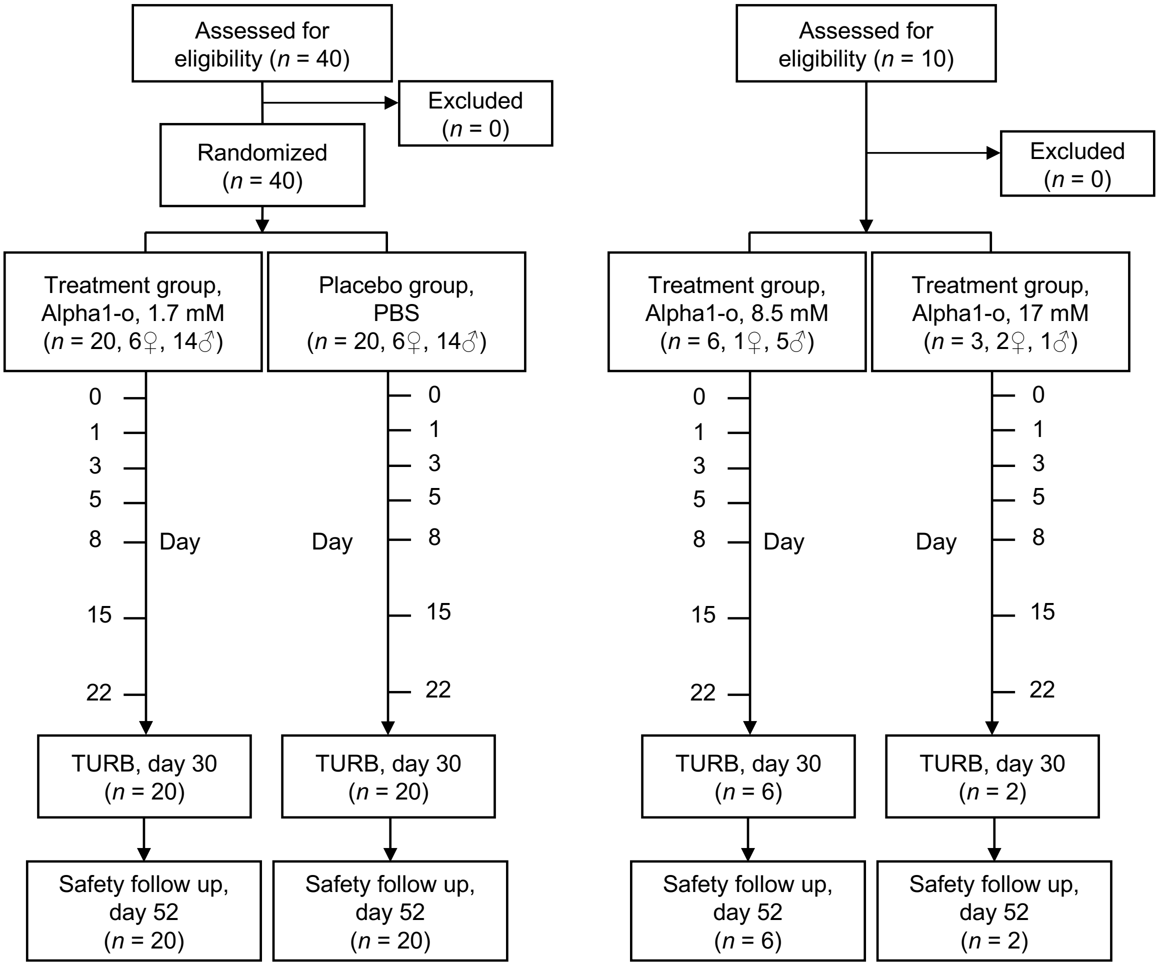

Fig. S1. Study CONSORT diagram.

Supplementary Figure 2

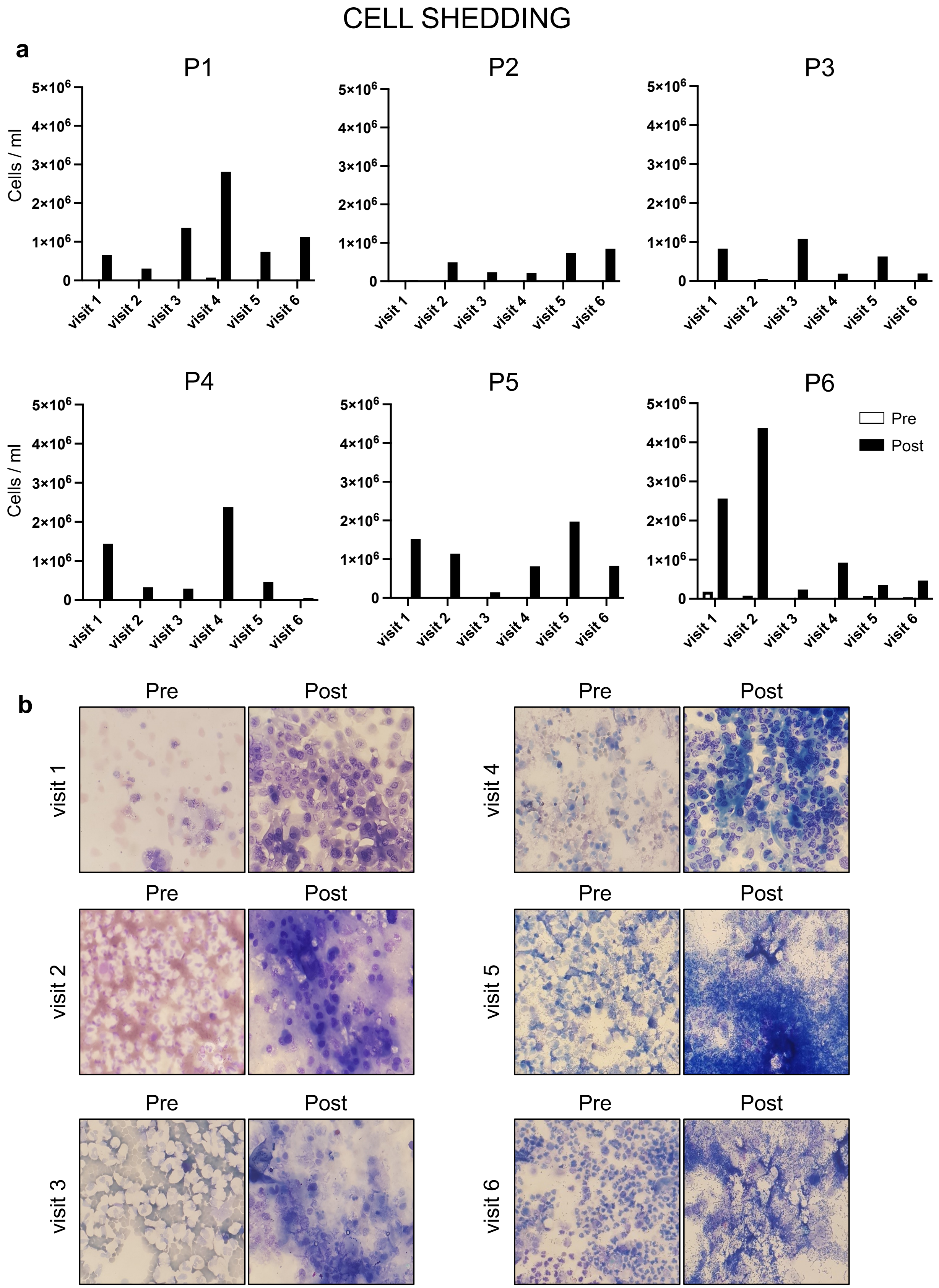

Fig. S2. Cell shedding into the urine in response to intra-vesical alpha1-oleate instillations. Cell shedding was quantified at each visit, before and approximately two hours after the instillation of 8.5 mM of alpha1-oleate. (**a**) Cell numbers in urine samples obtained pre (white) and post (black) alpha1-oleate instillation per patient. (**b**) Representative images illustrating the increase in cell shedding in pre compared to post instillation in different patients.

Supplementary Figure 3

a Changes in papillary stroma surface area (histo-pathology)

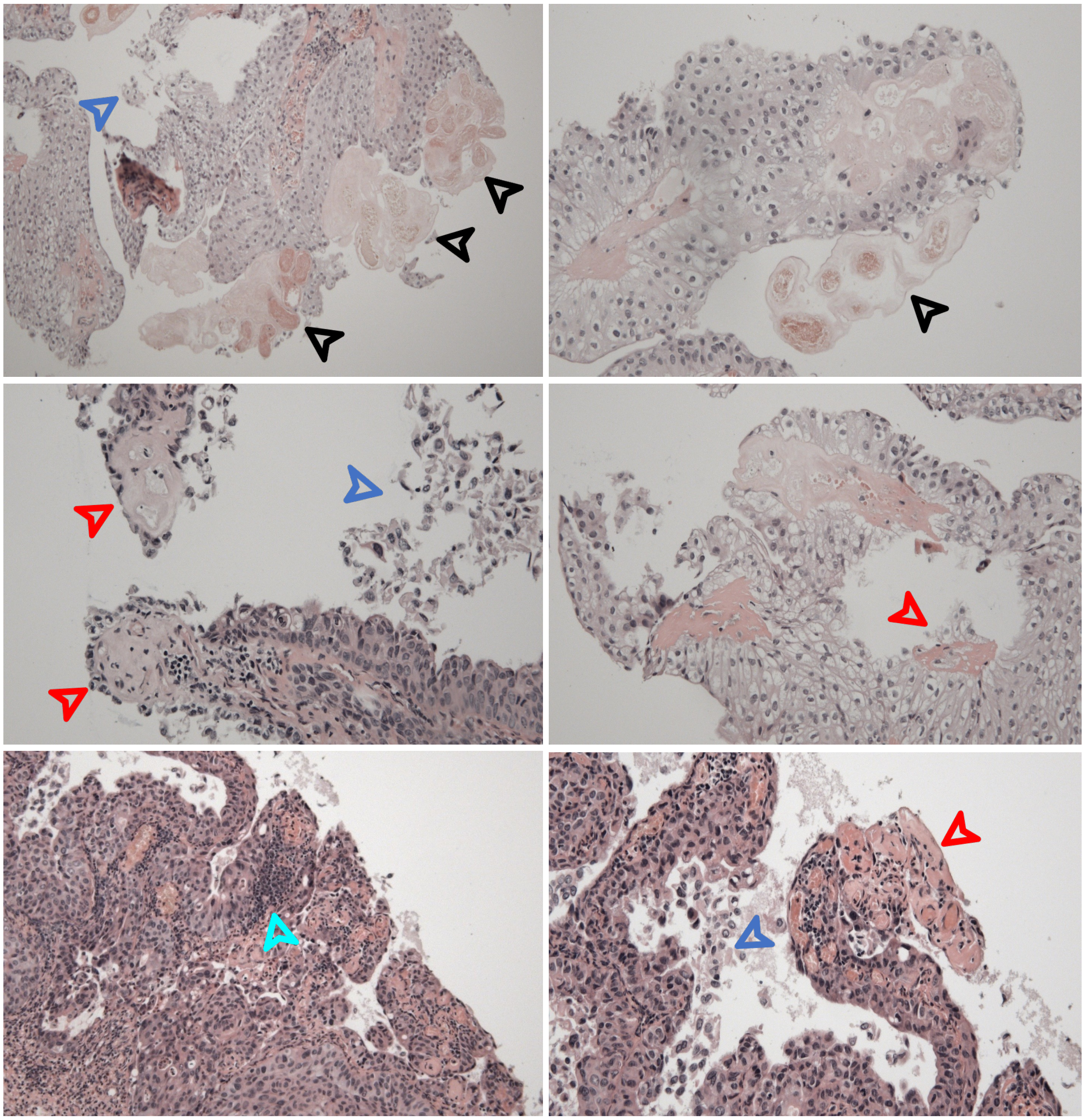

Naked papillary stroma · Cell desquamation · Inflammatory infiltrate · Necrosis

b

Summary of pathology evaluation	**Placebo N (%)**	**1.7 mM N (%)**	**8.5 mM N (%)**	**17 mM N (%)**
**Tumor grade (WHO 1973)**				
Missing	4 (20.0)			1 (50.0)
Papillary carcinoma, grade 1	6 (30.0)	4 (20.0)	4 (30.8)	
Papillary carcinoma, grade 2	7 (35.0)	14 (70.0)	7 (53.8)	
Papillary carcinoma, grade 3	3 (15.0)	2 (10.0)	2 (15.4)	1 (50.0)
**Tumor grade (WHO 2004/2016)**				
Missing	4 (20.0)			1 (50.0)
High-grade	4 (20.0)	5 (25.0)	3 (23.1)	1 (50.0)
Low-grade	12 (60.0)	15 (75.0)	10 (76.9)	
**Tumor stage**				
Ta	12 (75.0)	14 (70.0)	10 (76.9)	
T1	4 (25.0)	5 (25.0)	3 (23.1)	
T2	0	1 (5.0)	0	1 (50.0)

Fig. S3. Changes in papillary stroma surface area. (**a**) Histo-pathology of bladder tumor biopsies collected at the time of surgery in patients treated with alpha1-oleate 8.5 mM. Changes in papillary stroma surfaces are indicated by arrow heads, with epithelial cell desquamation (blue arrows), naked papillary stroma (red arrows), inflammatory infiltration of subepithelial stroma (cyan arrows) and papillary necrosis (black arrows). (**b**) Table indicating the Grade and Stage/Invasiveness of tumor biopsies using WHO scoring classifications.

Supplementary Figure 4

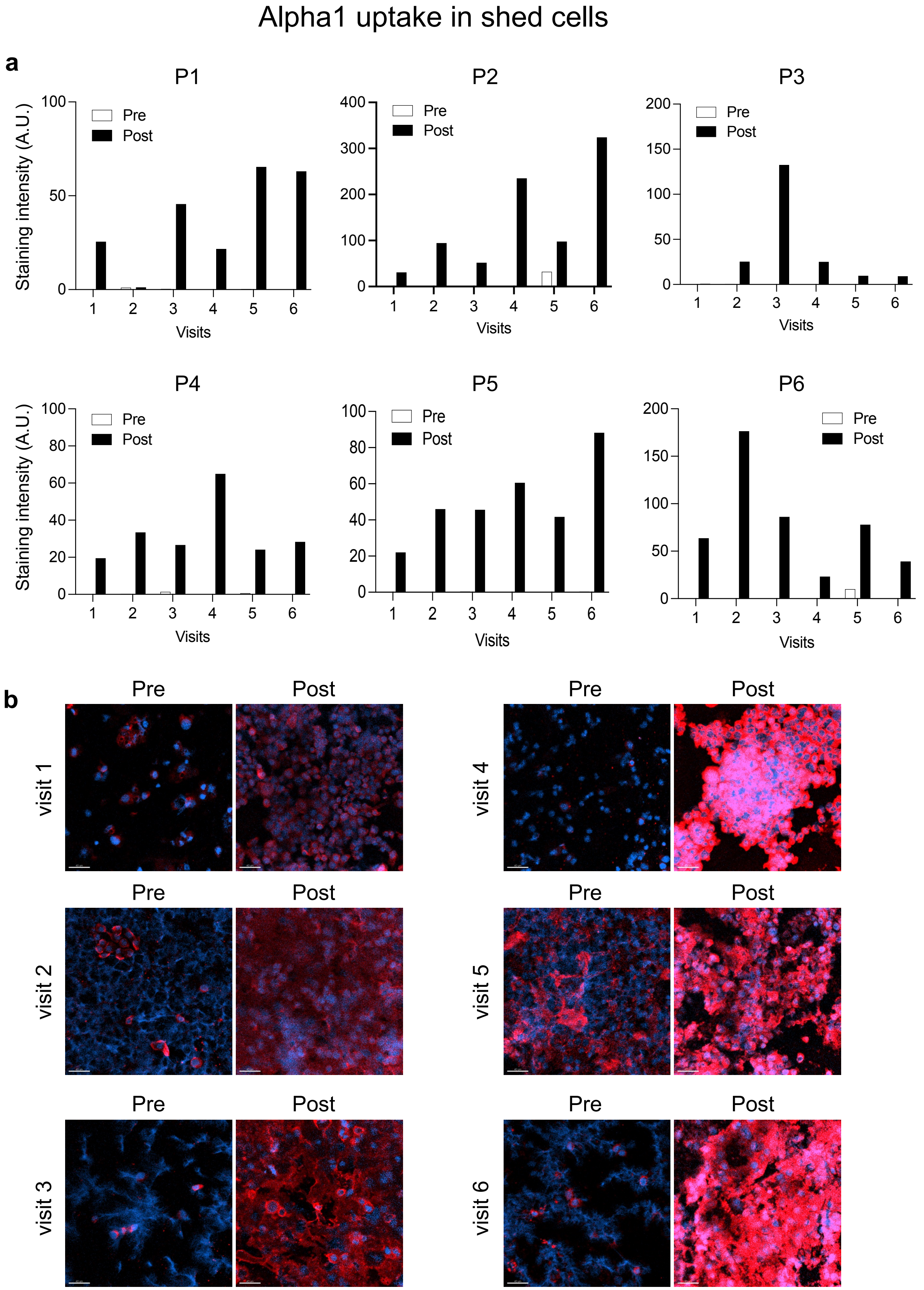

Fig. S4. Alpha1-oleate uptake in shed urine cells as a response to intra-vesical alpha1-oleate instillations. Alpha1-oleate uptake was quantified at each visit, before and approximately two hours. after the instillation of 8.5 mM of alpha1-oleate (**a**) Intensity of alpha1-oleate in urine samples obtained pre (white) and post (black) instillation per patient (**b**) Representative images illustrating the increase in alpha1 uptake in shed cells in post compared to pre instillation in different patients.

Supplementary Figure 5

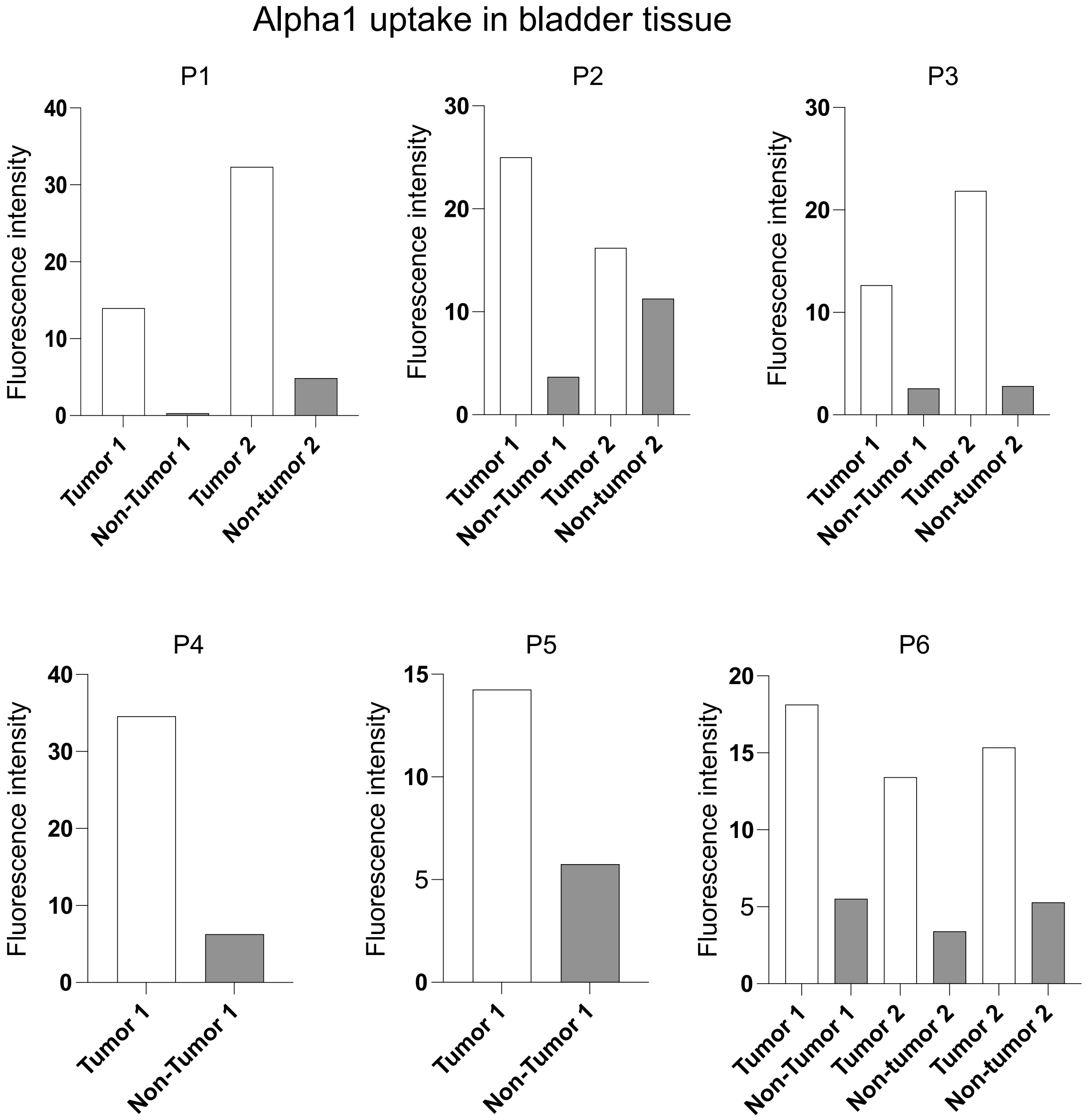

Fig. S5. Alpha1-oleate uptake in tumor tissue as a response to intra-vesical alpha1-oleate instillations. Alpha1 staining of tissue biopsies from patients treated with alpha1-oleate (8.5 mM). Increase in intensity of alpha1-oleate in tissues obtained from patients' tumor region compared to Non-tumor region in the same biopsy.

Supplementary Figure 6

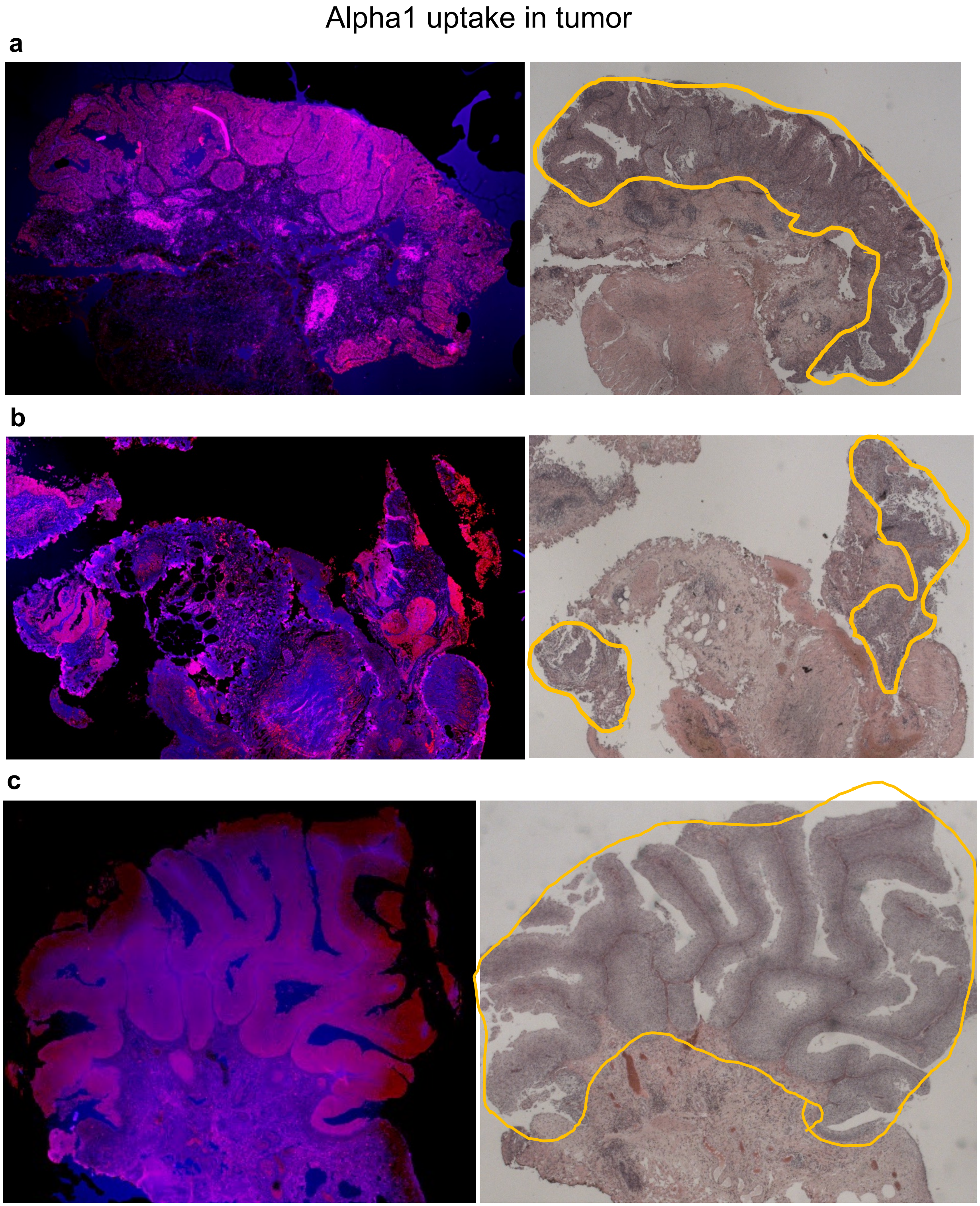

Fig. S6. Alpha1-oleate uptake in tumor tissue as a response to intra-vesical alpha1-oleate instillations. Left images indicate the alpha1-oleate staining, while the right images indicate the H and E staining with marked tumor region outlined with yellow line. (a-c) Representative images of increase in intensity of alpha1-oleate in tissues obtained from different patients. Red- alpha1; blue- nucleus.

Supplementary Figure 7

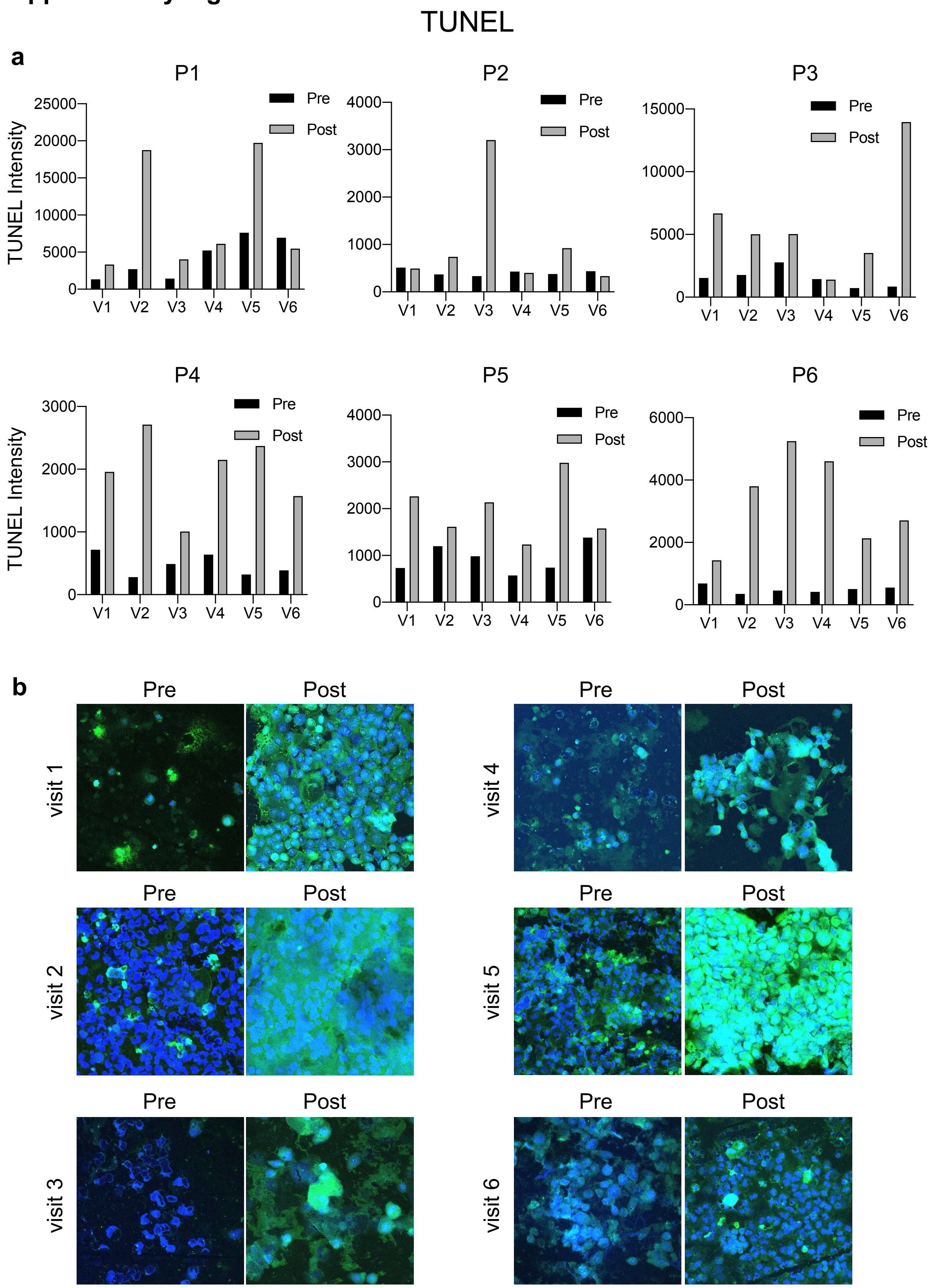

Fig. S7. TUNEL uptake in shed urine cells as a response to intra-vesical alpha1-oleate instillations. TUNEL uptake was quantified at each visit, before and approximately two hours. after the instillation of 8.5 mM of alpha1-oleate (**a**) Intensity of TUNEL in urine samples obtained pre (black) and post (grey) instillation per patient (**b**) Representative images illustrating the increase in TUNEL uptake in shed cells in post compared to pre instillation in different patients. (green = TUNEL, blue = nucleus)

Supplementary Figure 8

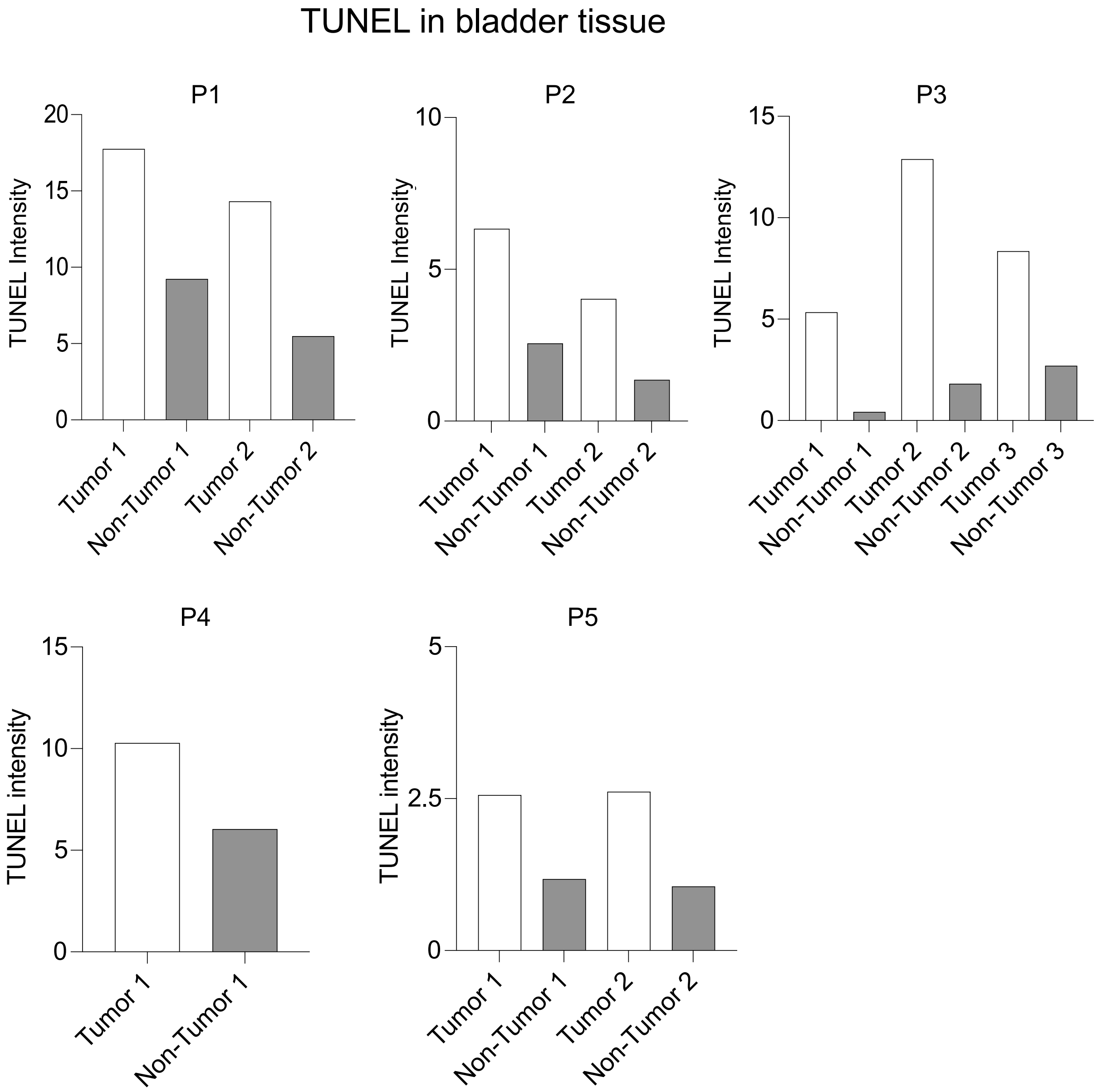

Fig. S8. TUNEL uptake in tumor tissue as a response to intra-vesical alpha1-oleate instillations. TUNEL staining of tissue biopsies from patients treated with alpha1-oleate (8.5 mM). Increase in intensity of TUNEL in tissues obtained from patients with Tumor region compared to Non-tumor region in the same biopsy.

Supplementary Figure 9

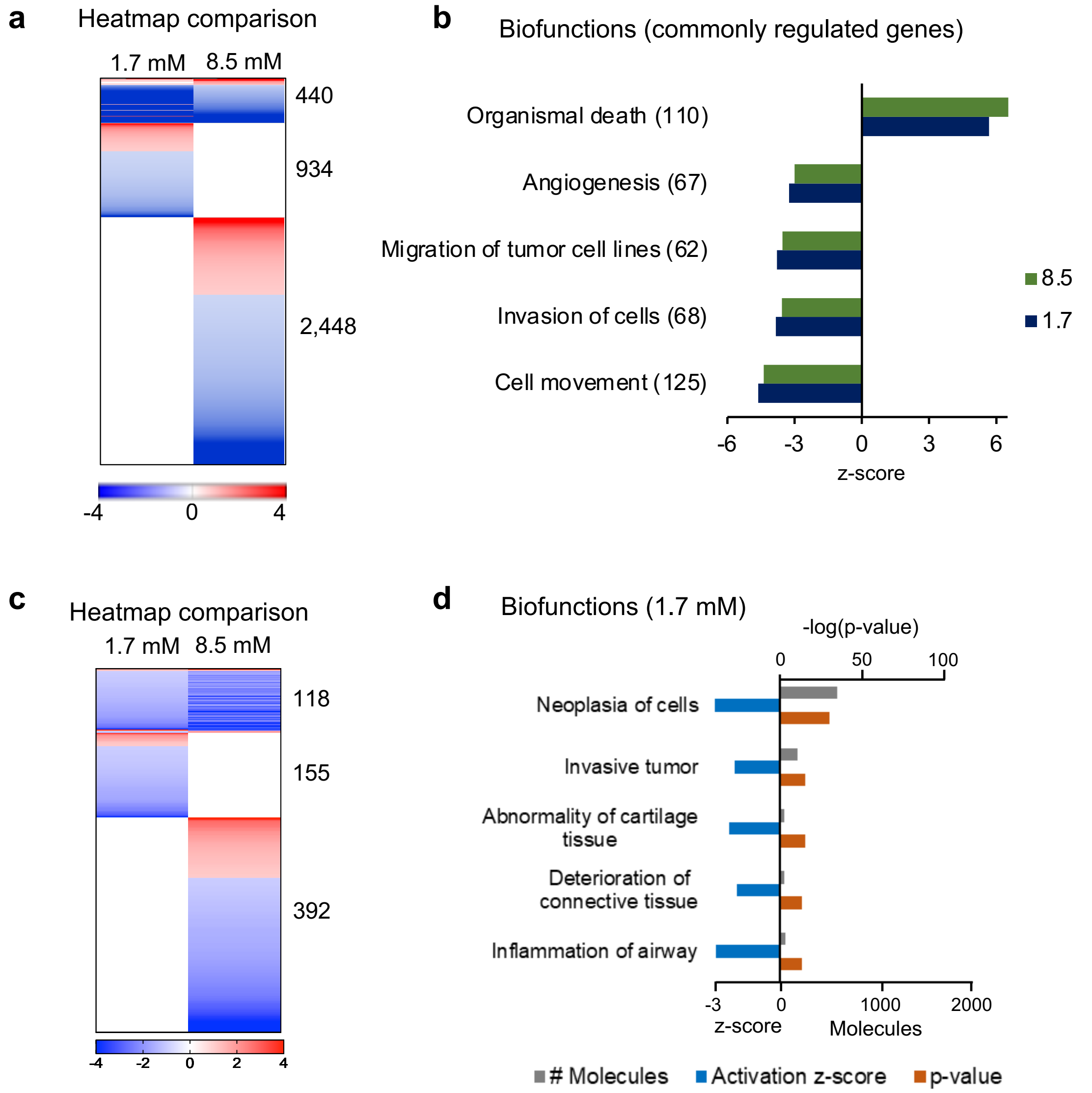

Fig. S9. Inhibition of cancer gene expression and cancer functions in treated tumors. (**a**) Heatmap comparing the gene expression response in the 1.7 mM and 8.5 mM treatment groups. 440 genes were commonly regulated, while 934 and 2,454 were specific to 1.7 mM and 8.5 mM, respectively. (**b**) Functional classification of the 440 genes commonly regulated between the 1.7 mM and 8.5 mM treatment groups. Apoptosis was activated and cancer-related genes inhibited (angiogenesis, tumor cell movement, migration and invasion) (z-score > 2, $P < 0.05$). (**c**) Heat map comparing the expression of apoptosis-related genes in the 1.7 mM and 8.5 mM treatment groups, compared to placebo. (**d**) Top regulated cancer associated functions in 1.7 mM treated tumors (blue = z-score, orange = *P*-value and grey = molecules). Inflammation, neoplasia, invasion, abnormality of cartilage tissue and deterioration of connective tissue were inhibited (z-score < -2, $P < 0.05$).

Supplementary Figure 10

a Heatmap of **HALLMARK_APOPTOSIS** related molecules

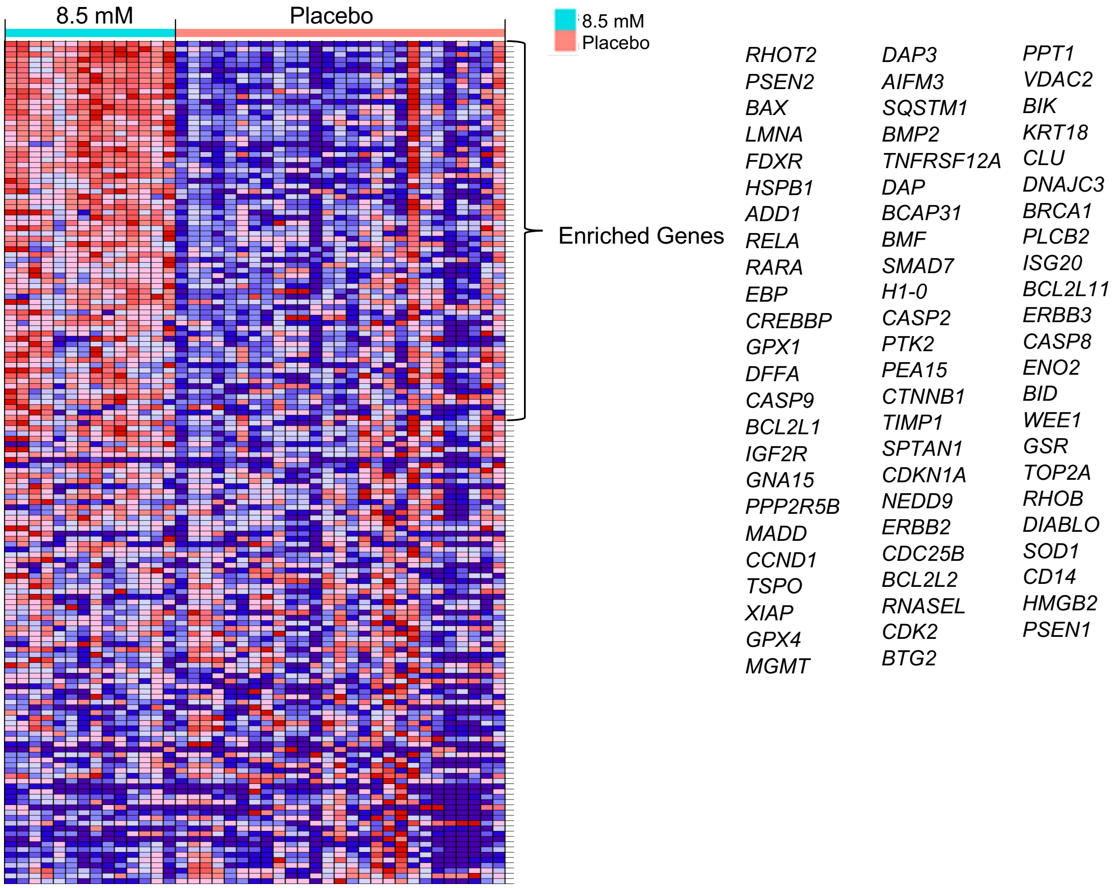

b Heatmap of **KEGG_APOPTOSIS** related molecules

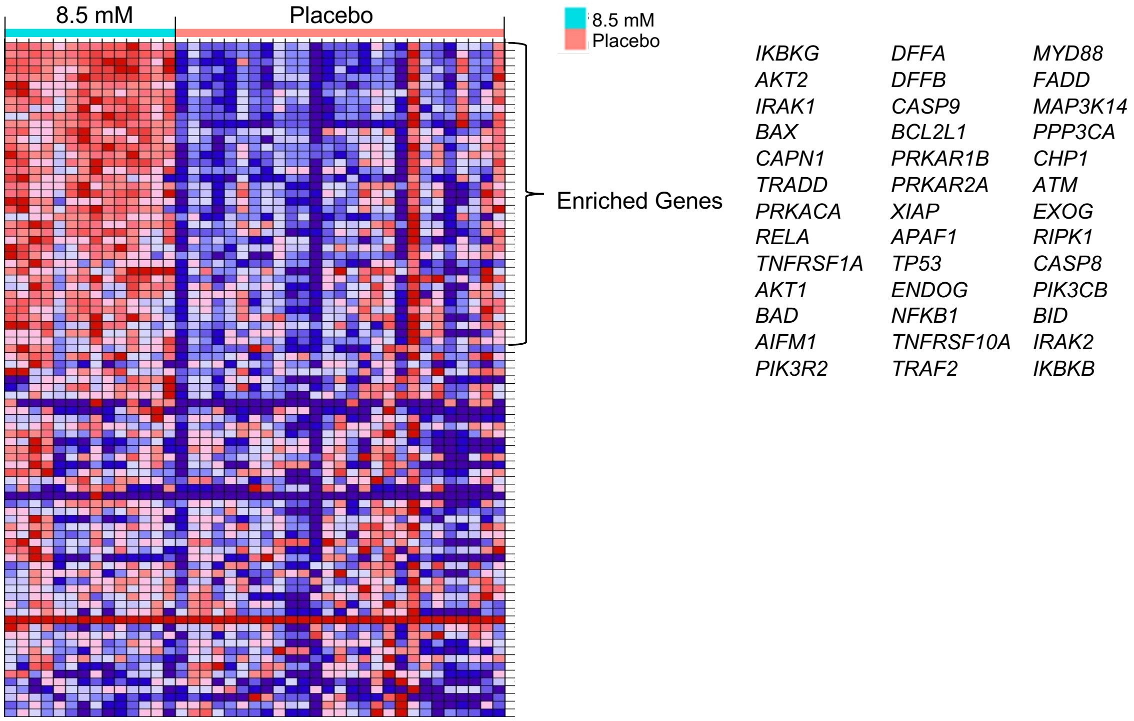

Fig. S10. GSEA analysis apoptosis of related gene sets in the 8.5 mM treatment group. (**a,b**) Heatmaps of gene expression of apoptosis-related genes in individual tumors samples. The list of genes enriched in the 8.5 mM group is shown for each gene set.
